# Chemo-Click: Receptor-Controlled and Bioorthogonal Chemokine Ligation for Real-Time Imaging of Drug-Resistant Leukemic B Cells

**DOI:** 10.1021/jacs.4c12035

**Published:** 2024-10-23

**Authors:** Marco Bertolini, Lorena Mendive-Tapia, Utsa Karmakar, Marc Vendrell

**Affiliations:** † Centre for Inflammation Research, The University of Edinburgh, Edinburgh EH16 4UU, U.K.; ‡ IRR Chemistry Hub, Institute for Regeneration and Repair, The University of Edinburgh, Edinburgh EH16 4UU, U.K.

## Abstract

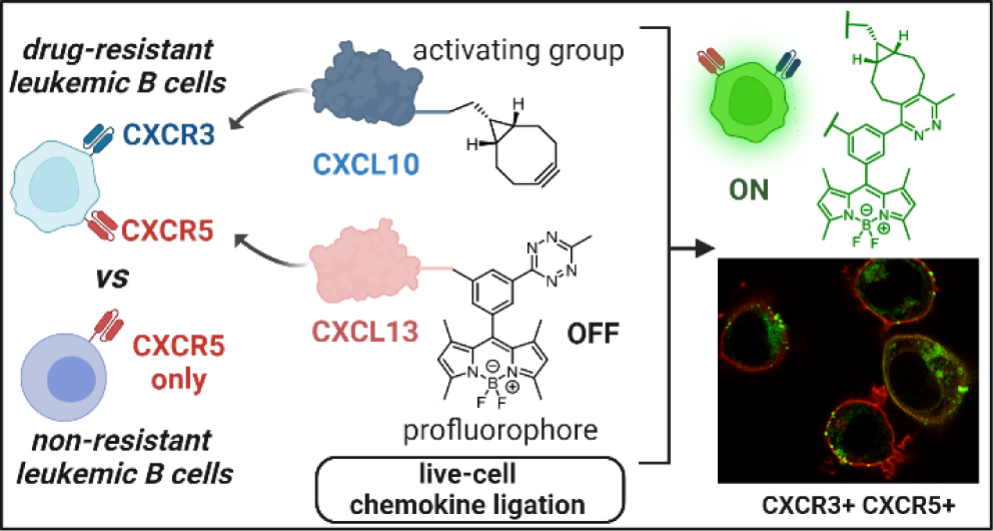

Drug resistance in B cell leukemia is characterized by the coexpression of CXCR5 and CXCR3 chemokine receptors, making it a valuable biomarker for patient stratification. Herein, we report a novel platform of activatable chemokines to selectively image drug-resistant leukemic B cells for the first time. The C-terminal derivatization of the human chemokines CXCL13 and CXCL10 with bioorthogonal tetrazine-BODIPY and BCN groups retained binding and internalization via their cognate CXCR5 and CXCR3 receptors and enabled rapid fluorescence labeling of CXCR5+ CXCR3+ resistant B cells—but not drug-susceptible leukemic cells—via intracellular chemokine ligation. This modular chemical approach offers a versatile strategy for real-time immunophenotyping of cell populations with distinct chemokine profiles and will accelerate the design of new precision medicine tools to advance personalized therapies in blood tumors.

## Introduction

B cell leukemia is one of the most common blood tumors,^1^ and a substantial fraction of patients experience drug resistance and relapse during treatment.^2,3^ Among the molecular features to discern between susceptible and resistant cancerous B cells, functional chemokine receptors hold potential as biomarkers for invasiveness and response to therapy.^4^ Specifically, the chemokine receptors CXCR3 and CXCR5, alongside their respective chemokine ligands CXCL10 and CXCL13, play essential roles in the migration of cancerous B cells in blood tumors.^5,6^ CXCR5 receptors are expressed in mature B cells (but not in other lymphocytes like CD8^+^ T cells),^7^ while CXCR3 receptors are directly associated with invasiveness and drug resistance.^8^ Despite the importance of these biomarkers, currently there are no chemical strategies to selectively target the distinct chemokine signatures of drug-resistant leukemic cells.

**Figure 1:**
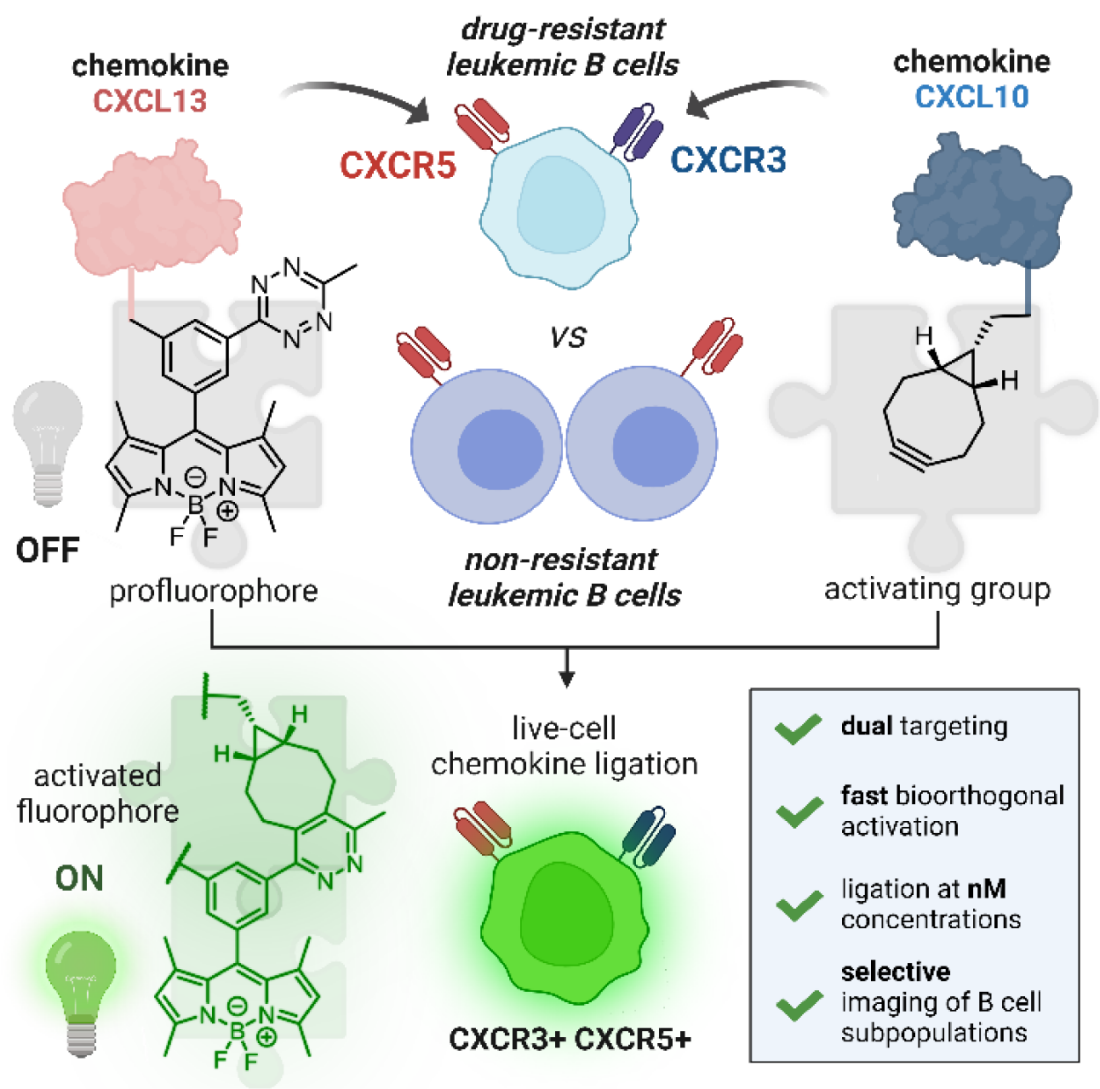
Schematic representation of receptor-controlled targeting of resistant leukemic B cells using bioorthogonal chemokine ligation. The chemokines CXCL10 and CXCL13 are modified with BODIPY fluorophores or activating groups. Because chemokines are recognized by their cognate receptors (e.g., CXCR3 and CXCR5, respectively), the fluorescent bioorthogonal adduct is only formed in B cells expressing both receptors (i.e., drug-resistant leukemic cells).

Dual-locked and AND-gate molecular designs are an emerging class of probes that simultaneously recognize two or more biomarkers to enhance target selectivity,^9−14^ such as enzyme-activatable substrates for cancer cells or tumor-associated leukocytes.^15−20^ Our group reported AND-gate fluorescent constructs for imaging immune cells, including metastasis-associated macrophages;^21,22^ however, these probes target a single class of cell-surface receptors and cannot report on drug resistance profiles. Some other strategies employ fluorophores binding to transporters or antigen receptors,^23−26^ but these are not specific for subpopulations of B cells. Given the close association between the coexpression of CXCR5 and CXCR3 receptors and drug resistance in leukemic B cells,^27^ we envisioned a novel imaging platform exploiting bioorthogonal chemokine ligation to selectively target resistant B cells expressing both CXCR5 and CXCR3 receptors (Figure 1).

Bioorthogonal strategies enable fluorescence labeling of subcellular organelles,^28,29^ glycans,^30^ nucleic acids,^31,32^ lipids^33^ and other metabolites.^34,35^ Some of these approaches build on the quenching ability of tetrazines and their selective activation with dienophiles.^36^ Tetrazine-containing probes have been reported in photoactivatable decaging and antibody targeting,^37,38^ but there are no fluorogenic tetrazines whose emission is spatially controlled by the expression of functional G-protein coupled receptors. In this work, we report a subpopulation-specific labeling approach using tandem chemokine activatable conjugates and demonstrate their application for real-time imaging of drug-resistant leukemic B cells.

## Results and Discussion

We started the design of this platform with the chemical synthesis of a fluorogen:activator pair that could react within minutes and emit fluorescence at the nanomolar concentrations needed for engagement with chemokine receptors in B cells. We selected boron dipyrromethenes (BODIPY) as bright and biocompatible fluorophores that can be modified with tetrazine moieties to modulate their optical properties.^39−41^ Tetrazine-modified BODIPYs are quenched via resonance and through-bond energy transfer,^42,43^ and their fluorescence can be restored by inverse electron-demand Diels–Alder reactions with dienophiles.

**Figure 2:**
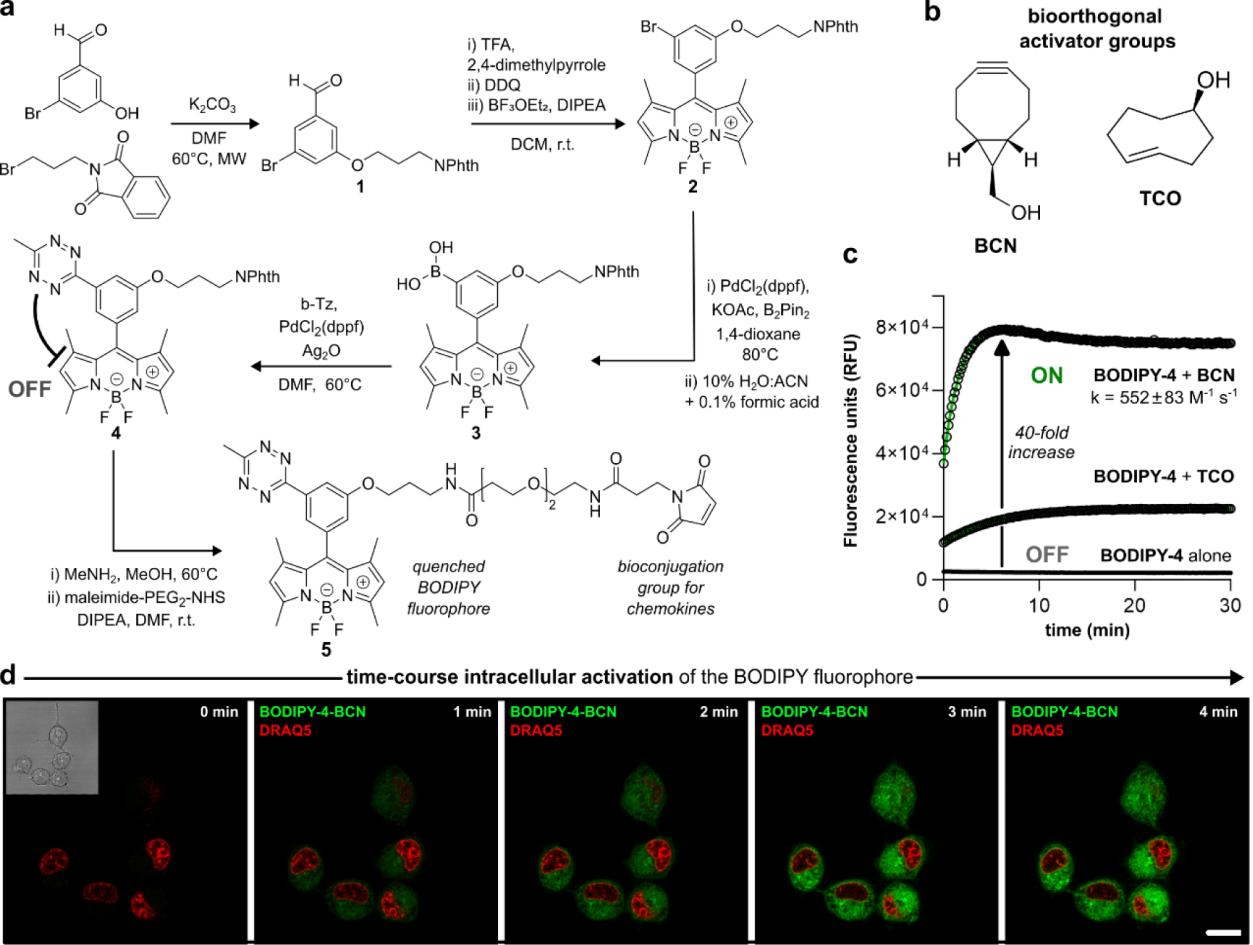
Synthesis of a BODIPY fluorogen-activator pair for chemokine ligation and live-cell imaging. (a) Synthesis of the fluorogenic BODIPY **5** bearing a methyltetrazine quencher and a PEG-maleimide handle for bioconjugation. (b) Structures of endo-9-hydroxymethyl-bicyclo[6.1.0]non-4-yne (BCN) and trans-cyclooctenol (TCO) as tetrazine-reactive groups. (c) Time-dependent fluorescence emission of tetrazine-BODIPY **4** (10 μM, λ_exc_: 480 nm, λ_em_: 510 nm) before and after reaction with BCN or TCO (1 mM) (*n* = 3). (d) Time-course fluorescence microscopy images of MCF-7 cells after incubation with compound **4** (10 μM, green) and addition of BCN (100 μM). Movie S1. Cell nuclei were counterstained with DRAQ5 (5 μM, red). Scale bar: 10 μm.

We designed a synthetic scheme to functionalize BODIPY fluorophores with 1) methyltetrazine groups for intramolecular quenching, and 2) a maleimide polyethylene glycol (PEG) handle for bioconjugation to chemokines^44,45^ (Figure 2a). We alkylated 3-bromo-5-hydroxybenzaldehyde to obtain the intermediate compound **1** and used it in a standard condensation to isolate the BODIPY compound **2** with yields around 60%. Next, we introduced a boronic acid group in the BODIPY core by Miyaura borylation followed by hydrolysis in acidic conditions to afford compound **3**. Compound **3** was subjected to a modified Liebeskind-Srogl cross-coupling to incorporate a methyltetrazine quencher (i.e., tetrazine BODIPY **4**), and selective removal of the phthalimide protecting group and reaction with a maleimide-functionalized PEG spacer yielded the final BODIPY **5** as a fluorogenic reagent for chemokine derivatization (synthetic and characterization details in the Supporting Information.)

Next, we investigated the kinetics and fluorescence response of tetrazine BODIPYs after reaction with two commercially available dienophiles, namely trans-cyclooctenol (TCO, Figure 2b) and endo-9-hydroxymethyl-bicyclo[6.1.0]non-4-yne (BCN, Figure 2b). In these experiments, we monitored the reaction between compound **4** and the TCO or BCN activator groups by fluorescence analysis (Figure 2c) and HPLC-MS (Figure S1). Both reactions proceeded quickly and rendered the expected conjugation products with complete conversions at short time points (i.e., under 15 min) (Figure S1). Specifically, the reaction between the tetrazine-BODIPY **4** and BCN led to higher fluorescence enhancements (e.g., around 40-fold, Figure 2c), indicating the suitability of the pair **4**-BCN as a labeling strategy. Additional photophysical assays confirmed substantial differences in fluorescence quantum yields (i.e., ∼ 2% for the quenched BODIPY **4** and >90% upon formation of the adduct with BCN, Figure S2). We also analyzed the kinetics of the reaction between compound **4** and BCN and observed a pseudo-first-order rate constant of 5 × 10^2^ M^−1^s^−1^ (Figure S3), which is in agreement with previous reports of tetrazine probes.^46^

Lastly, we examined the suitability of the tetrazine **4**-BCN labeling pair for intracellular activation using time-course fluorescence microscopy in live cells. In these experiments, we first incubated MCF-7 cancer cells with compound **4**, which did not emit any detectable fluorescent signals, and subsequently added the cell-permeable dienophile BCN to monitor the formation of the fluorescent adduct without any washing steps. As shown in Figure 2d and in Movie S1, we observed bright intracellular signals in less than 5 min, confirming that fluorogenic tetrazine-BODIPYs can be activated inside cells with high signal-to-noise (S/N) ratios (i.e., S/N values >350). Furthermore, this bioorthogonal labeling pair showed no significant cytotoxicity when incubating the cells with compound **4**, BCN or the adduct **4**-BCN (Figure S4).

**Figure 3:**
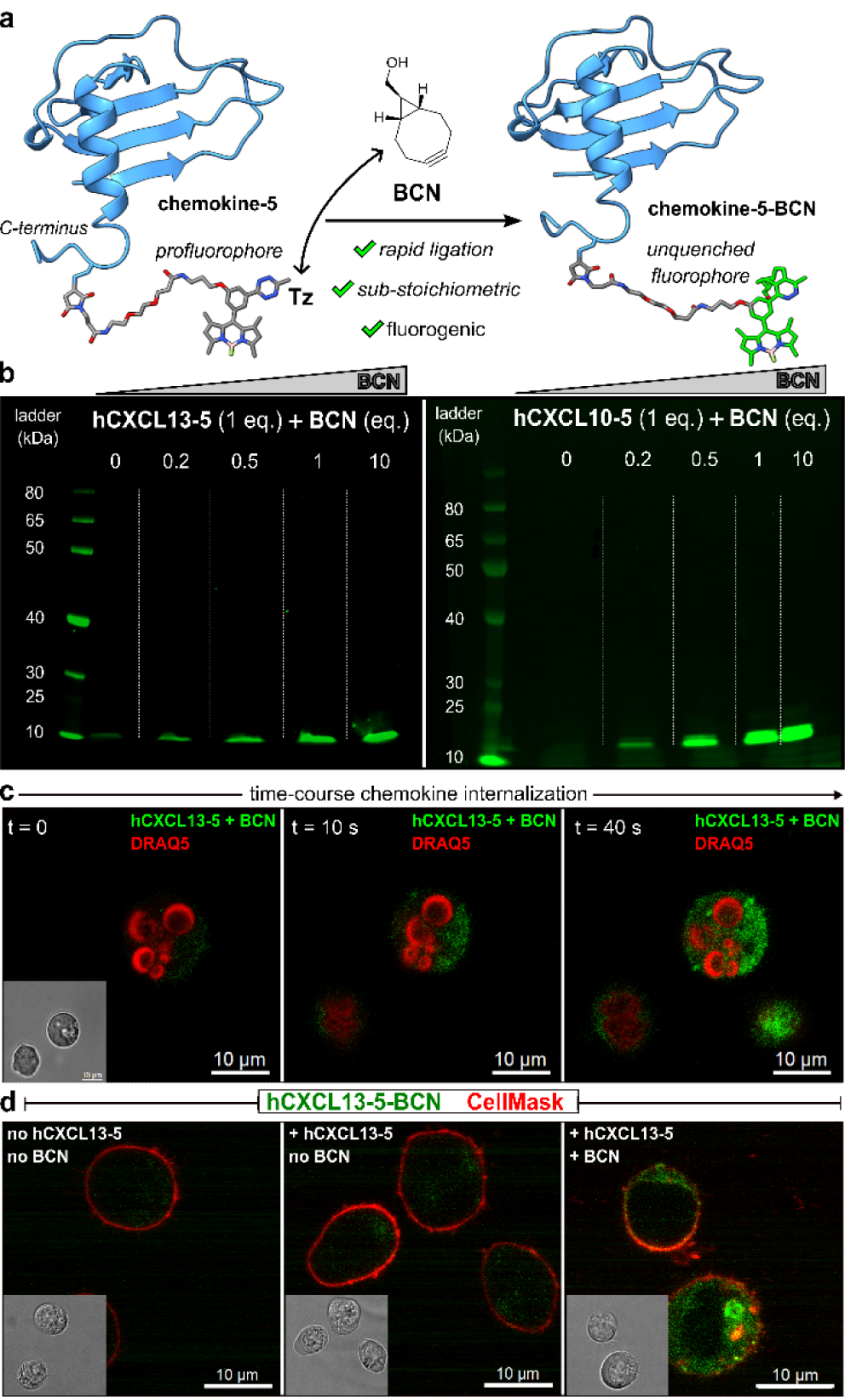
Fluorogenic chemokines are rapidly activated inside human B cells. (a) Schematic illustration of tetrazine-BODIPY chemokines and their bioorthogonal activation with BCN (PDB code: 7JNY). (b) Representative SDS-PAGE gels of **hCXCL13-5** (left) and **hCXCL10-5** (right) after reaction with increasing amounts of BCN (λ_exc_: 488 nm, λ_em_: 520 nm). (c) Time-course fluorescence microscopy images of CXCR5-expressing Raji cells after incubation with **hCXCL13-5** (500 nM, green) and nuclear counterstain DRAQ5 (5 μM, red) followed by BCN (100 equiv). Scale bars: 10 μm. Excitation wavelengths: 488 nm (**hCXCL13-5**), 633 nm (DRAQ5). Movie S2. (d) Brightfield and fluorescence microscopy images of Raji cells after incubation with **hCXCL13-5** (500 nM, green) and CellMask Deep Red (1:2000 dilution, red) with and without BCN (100 equiv). Scale bar: 10 μm. Excitation wavelengths: 488 nm (**hCXCL13-5**), 660 nm (CellMask Deep Red).

Having identified an optimal bioorthogonal pair for in situ monitoring of biomolecular ligation, we next proceeded to conjugate both human chemokines CXCL13 (hCXCL13) and CXCL10 (hCXCL10) to the maleimide-functionalized tetrazine-BODIPY **5**. Because both hCXCL13 and hCXCL10 chemokines interact with their respective CXCR5 and CXCR3 receptors via the N-terminal regions,^47,48^ we used chemokines containing one additional Cys residue at the C-terminus for site-specific coupling to compound **5** (Figure 3a). The conjugations of hCXCL13 and hCXCL10 to compound **5** were performed in PBS (10 mM, pH 7), and the two **hCXCL13-5** and **hCXCL10-5** labeled chemokines were purified by ultracentrifugation and analyzed by mass spectrometry (Figure S5).

We examined the reactivity of the tetrazine groups in **hCXCL13-5** and **hCXCL10-5** by titrating the chemokines with increasing concentrations of BCN, from subequimolar to excess amounts (i.e., 0.2 to 10 equiv) (Figure 3b). All reactions were performed in PBS at r.t. for 30 min, and the products were analyzed by SDS-PAGE and in-gel fluorescence scanning (Figure 3b). In both cases, in-gel analysis confirmed that the fluorescence intensity was directly proportional to the concentration of BCN and to the formation of bright adducts ∼10 kDa matching the labeled chemokines **hCXCL13-5-BCN** and **hCXCL10-5-BCN** (Figures S6 and S7). This result confirmed that 1) the reactivity of the tetrazine group of BODIPY **5** was retained after conjugation to the chemokine ligands, and 2) bioorthogonal activation could take place even when substoichiometric concentrations of BCN were used.

Next, we examined the binding, internalization and activation of the chemokine **hCXCL13-5** in B cells expressing CXCR5 receptors. For this purpose, we employed the human B-cell lymphoma line Raji as an exemplar of drug-susceptible cancerous B cells.^49^ Raji cells express CXCR5 receptors, as confirmed by staining them with a commercial anti-hCXCR5 antibody (Figure S8). Flow cytometry analysis showed that **hCXCL13-5** stained Raji cells at nanomolar concentrations, which is consistent with the reported affinity for CXCR5 (Figure S9).^50^ Next, we incubated Raji cells with **hCXCL13-5** (500 nM) followed by an excess of BCN under constant imaging by confocal microscopy (Figure 3c and Movie S2). Fluorescence microscope images confirmed bright signals only in cells that had been incubated with both **hCXCL13-5** and BCN but not in cells that had been only treated with **hCXCL13-5** (Figure 3d). Similarly, we verified the fluorescence activation of **hCXCL10-5** in CXCR3-expressing B cells. As expected, the coincubation of **hCXCL10-5** and BCN -but not with the individual reagents- resulted in bright intracellular signals (Figure S10). Altogether, these results corroborate the reactivity of tetrazine-quenched chemokines at nM concentrations and their suitability for real-time imaging of B cells expressing differential chemokine receptors.

Having confirmed in cellulo activation of the fluorogenic chemokines **hCXCL13-5** and **hCXCL10-5** with BCN as a nontargeted activator, we prepared their complementary BCN-containing chemokine conjugates for tandem ligation in leukemic B cells. Briefly, the two chemokines hCXCL13 and hCXCL10 were reacted with BCN-PEG_3_-maleimide, and the resulting **hCXCL13-BCN** and **hCXCL10-BCN** conjugates were purified by ultracentrifugation and analyzed by mass spectrometry (Figure S11). With all four chemokine conjugates in hand, next we investigated their behavior in drug-resistant leukemic B cells. For these experiments, we employed WSU-NHL cells, which are derived from a B-cell leukemia patient with refractory disease.^51^ Unlike Raji cells, which do not express high levels of CXCR3 receptors (Figure S12), WSU-NHL cells express both CXCR5 and CXCR3 receptors, which we corroborated by flow cytometry (Figure S13) and qPCR analysis (Figure S14). First, we measured the chemotactic ability of all chemokines (i.e., **hCXCL10-5**, **hCXCL10-BCN**, **hCXCL13-5** and **hCXCL13-BCN**) and compared them to the unlabeled analogues (i.e., hCXCL10 and hCXCL13). For these experiments, WSU-NHL cells were cultured on permeable membranes, and we measured their migration in response to different chemokine gradients. All functionalized chemokines induced similar migration to the native ligands, confirming retention of functional activity (Figure S15). These results confirmed that the C-terminal modification of hCXCL10 and hCXCL13 with tetrazine-BODIPY or BCN groups did not impair recognition of the cognate receptors. Next, we assessed the ability of the chemokine conjugates to undergo rapid fluorogenic ligation in a receptor-controlled manner. For this purpose, the bioorthogonally paired chemokines **hCXCL13-5** and **hCXCL10-BCN** were combined at equimolar concentrations for 30 min, and the reaction products were analyzed by SDS-PAGE and in-gel fluorescence scanning. Notably, the reaction mixtures displayed bright fluorescent bands at 20 kDa corresponding to the chemokine ligation product (Figure S16).

**Figure 4:**
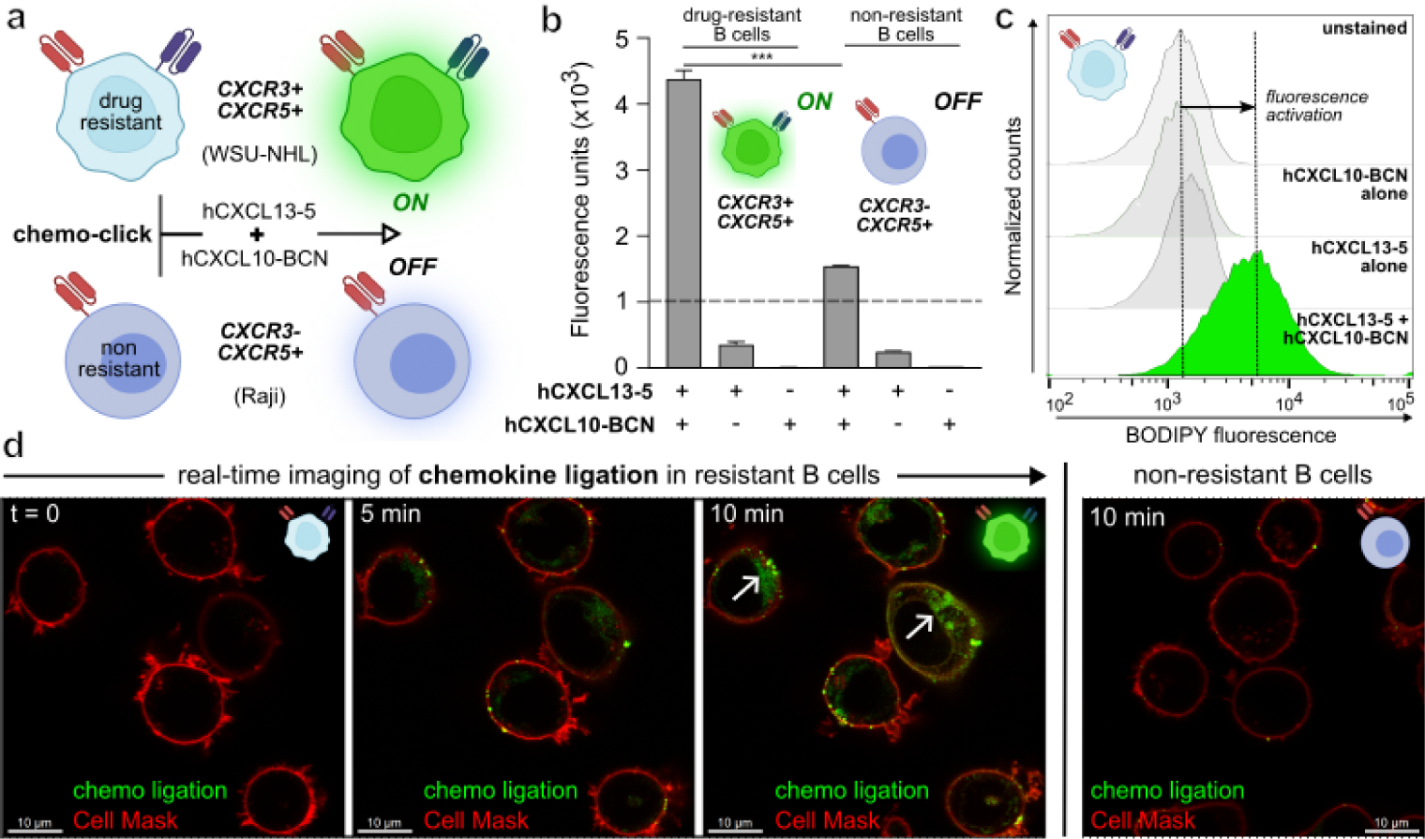
The combined bioorthogonal pair **hCXCL13-5** and **hCXCL10-BCN** selectively labels drug-resistant leukemic B cells over nonresistant B cells. (a) Schematic representation of receptor-controlled fluorogenic ligation of **hCXCL13-5** and **hCXCL10-BCN** in resistant WSU-NHL cells over nonresistant Raji cells. (b) Fluorescence emission of drug-resistant B cells (CXCR3+CXCR5+) and nonresistant B cells (CXCR3–CXCR5+) after addition of **hCXCL13-5** (220 nM) and **hCXCL10-BCN** (290 nM). Data as means ± SD (*n* = 3). *** for *p* < 0.001. (c) Representative histograms showing fluorescence activation in WSU-NHL cells. (d) Real-time imaging of chemokine ligation inside leukemic B cells (white arrows) after addition of **hCXCL10-BCN** (1 μM) and **hCXCL13-5** (440 nM, green). Movies S3 (WSU-NHL) and S4 (Raji). Cells were costained with CellMask Deep Red (1:2000 dilution, red) as a membrane marker. Excitation wavelengths: 488 nm (**hCXCL13-5**), 660 nm (CellMask Deep Red). Scale bar: 10 μm.

We also investigated the utility of the receptor-controlled ligation platform to selectively label drug-resistant (WSU-NHL cells) over nonresistant leukemic B cells (Raji cells) (Figure 4a). Both cells were first incubated with **hCXCL13-5** for 30 min at 37 °C, washed and subsequently incubated with **hCXCL10-BCN** for 30 min. Flow cytometry experiments showed that the treatment with both **hCXCL13-5** and **hCXCL10-BCN** resulted in clear differential staining between nonresistant Raji B cells (i.e., weakly fluorescent) and drug-resistant WSU-NHL cells (i.e., highly fluorescent) (Figure 4b,c). Importantly, control experiments with single chemokines showed marginal levels of fluorescence emission in both subsets of B cells (Figures 4b and S17). Additional experiments in WSU-NHL cells with the alternate bioorthogonal chemokine pair (i.e., **hCXCL13-BCN** and **hCXCL10-5**) showed comparable staining, indicating that the order of chemokine addition was not critical for the bioorthogonal ligation (Figure S18), and fluorescent signals were reduced when WSU-NHL cells were preincubated with an anti-hCXCR3 antibody (Figure S19). We also assessed the real-time imaging capabilities of the bioorthogonal chemokine ligation. We performed time-course flow cytometry analysis in drug-resistant WSU-NHL cells that had been incubated with **hCXCL13-5** (200 nM) for 30 min at 37 °C, followed by a washing step and addition of the **hCXCL10-BCN** counterpart (250 nM). WSU-NHL cells displayed bright fluorescence emission in minutes, which confirms the utility of this labeling strategy for rapid fluorescence-based assays (Figure S20). We also corroborated these findings by live-cell microscopy where the coaddition of **hCXCL13-5** and **hCXCL10-BCN** led to bright intracellular staining of drug-resistant WSU-NHL B cells (Figure 4d and Movie S3) unlike nonresistant Raji B cells (Figures 4d and S21 and Movie S4). Finally, we tested the selectivity of the chemokine combination in a mixed coculture of WSU-NHL and Raji cells, where fluorescent signals were only observed in WSU-NHL cells but not in Raji cells (Figure S22). Altogether, these results feature the high selectivity and sensitivity of receptor-controlled chemokine ligation to distinguish between subpopulations of leukemic B cells using bioorthogonal chemistry and fluorescence-based assays. Furthermore, the modularity and versatility of this labeling strategy with additional biomarkers (e.g., other chemokines, growth factors) holds potential to generate new fluorescence-based technologies that can advance the prognosis and personalized treatment of blood tumors.

## Conclusions

In summary, we designed a new bioorthogonal imaging platform that builds on rapid chemokine ligation to target for the first time subpopulations of drug-resistant leukemic B cells. We have optimized a tetrazine-BODIPY fluorogen:BCN activator pair that reaches 40-fold fluorescence amplification in minutes and microscopy S/N ratios >350 for wash-free imaging. We introduced these groups at the C-terminal ends of the human chemokines **hCXCL13** (cognate ligand of CXCR5) and **hCXCL10** (cognate ligand of CXCR3) and demonstrated that the resulting conjugates retain chemotactic functionality and enable time-course flow cytometry and confocal microscopy in leukemic B cells. Notably, the **hCXCL13-5** and **hCXCL10-BCN** combo exhibits fast and selective intracellular labeling of drug-resistant B cells but not drug-susceptible ones, proving its utility to differentiate between close populations of immune cells in real time. The modularity of this tandem ligation strategy can be expanded to other immune cells with distinct chemokine signatures and to other receptor pairs (e.g., G-protein coupled receptors, growth factors), paving the way for new live-cell immunophenotyping technologies with enhanced spatiotemporal resolution. Furthermore, this platform could be in principle adapted to click-and-release strategies^52^ to enable precise delivery of therapeutic payloads to subpopulations of tumor cells.

## Supporting Information Available

Full synthetic and chemical characterization details; NMR spectra; experimental procedures; HPLC analysis (Figure S1); representative fluorescence spectra of compound **4** (Figure S2); time-lapse fluorescence emission of compound **4** (Figure S3); viability assays of MCF-7 cells incubated with compound **4** (Figure S4); mass spectrometry analysis (Figure S5); representative fluorescence intensity quantification and Coomassie stained SDS-PAGE (Figures S6 and S7); fluorescence histograms of Raji cells (Figures S8 and S9); representative confocal microscopy images (Figure S10); mass spectrometry analysis (Figure S11); fluorescence histograms of Raji and WSU-NHL cells (Figures S12 and S13); relative mRNA expression (Figure S14); transwell migration assays (Figure S15); representative SDS-PAGE gel (Figure S16); dual chemokine ligation in Raji cells (Figure S17); flow cytometry analysis (Figures S18 and S19); time-course chemokine ligation analysis (Figure S20); representative time-course fluorescence microscopy images (Figure S21) (PDF)
Time-lapse fluorogenic intracellular activation of compound **4** (10 μM, green) in MCF-7 cells after addition of excess BCN (100 μM) (AVI)
Time-lapse fluorescence microscopy of Raji cells after incubation with **hCXCL13-5** (500 nM, green) followed by addition of BCN (50 μM) (AVI)
Time-lapse fluorescence microscopy of WSU-NHL cells after incubation with **hCXCL13-5** (440 nM, green) followed by addition of **hCXCL10-BCN** (1 μM) (AVI)
Time-lapse fluorescence microscopy of Raji cells after incubation with **hCXCL13-5** (440 nM, green) followed by addition of **hCXCL10-BCN** (1 μM) (AVI)

## Supplementary Material

ja4c12035_si_001.pdf

ja4c12035_si_002.avi

ja4c12035_si_003.avi

ja4c12035_si_004.avi

ja4c12035_si_005.avi

## References

[ref1] Malard F.; Mohty M. Acute Lymphoblastic Leukaemia. Lancet 2020, 395 (10230), 1146–1162. 10.1016/S0140-6736(19)33018-1. 32247396

[ref2] Hunger S. P.; Mullighan C. G. Acute Lymphoblastic Leukemia in Children. N. Engl. J. Med. 2015, 373 (16), 1541–1552. 10.1056/NEJMra1400972. 26465987

[ref3] Toft N.; Birgens H.; Abrahamsson J.; Griškevičius L.; Hallböök H.; Heyman M.; Klausen T. W.; Jónsson Ó.; Palk K.; Pruunsild K.; Quist-Paulsen P.; Vaitkeviciene G.; Vettenranta K.; Åsberg A.; Frandsen T. L.; Marquart H. V.; Madsen H. O.; Norén-Nyström U.; Schmiegelow K. Results of NOPHO ALL2008 Treatment for Patients Aged 1–45 Years with Acute Lymphoblastic Leukemia. Leukemia 2018, 32 (3), 606–615. 10.1038/leu.2017.265. 28819280

[ref4] Trentin L.; Agostini C.; Facco M.; Piazza F.; Perin A.; Siviero M.; Gurrieri C.; Galvan S.; Adami F.; Zambello R.; Semenzato G. The Chemokine Receptor CXCR3 Is Expressed on Malignant B Cells and Mediates Chemotaxis. J. Clin. Invest. 1999, 104 (1), 115–121. 10.1172/JCI7335. 10393705 PMC408409

[ref5] Bürkle A.; Niedermeier M.; Schmitt-Gräff A.; Wierda W. G.; Keating M. J.; Burger J. A. Overexpression of the CXCR5 Chemokine Receptor, and Its Ligand, CXCL13 in B-Cell Chronic Lymphocytic Leukemia. Blood 2007, 110 (9), 3316–3325. 10.1182/blood-2007-05-089409. 17652619

[ref6] Jones D.; Benjamin R. J.; Shahsafaei A.; Dorfman D. M. The Chemokine Receptor CXCR3 Is Expressed in a Subset of B-Cell Lymphomas and Is a Marker of B-Cell Chronic Lymphocytic Leukemia. Blood 2000, 95 (2), 627–632. 10.1182/blood.V95.2.627. 10627472

[ref7] Förster R.; Emrich T.; Kremmer E.; Lipp M. Expression of the G-protein--coupled receptor BLR1 defines mature, recirculating B cells and a subset of T-helper memory cells. Blood 1994, 84 (3), 830–840. 10.1182/blood.V84.3.830.bloodjournal843830. 7913842

[ref8] Gómez A. M.; Martínez C.; González M.; Luque A.; Melen G. J.; Martínez J.; Hortelano S.; Lassaletta Á.; Madero L.; Ramírez M. Chemokines and Relapses in Childhood Acute Lymphoblastic Leukemia: A Role in Migration and in Resistance to Antileukemic Drugs. Blood Cells. Mol. Dis. 2015, 55 (3), 220–227. 10.1016/j.bcmd.2015.07.001. 26227851

[ref9] Wu L.; Huang J.; Pu K.; James T. D. Dual-Locked Spectroscopic Probes for Sensing and Therapy. Nat. Rev. Chem. 2021, 5 (6), 406–421. 10.1038/s41570-021-00277-2. 37118027

[ref10] Scott J. I.; Deng Q.; Vendrell M. Near-Infrared Fluorescent Probes for the Detection of Cancer-Associated Proteases. ACS Chem. Biol. 2021, 16 (8), 1304–1317. 10.1021/acschembio.1c00223. 34315210 PMC8383269

[ref11] Mendive-Tapia L.; Vendrell M. Activatable Fluorophores for Imaging Immune Cell Function. Acc. Chem. Res. 2022, 55 (8), 1183–1193. 10.1021/acs.accounts.2c00070. 35380423 PMC9022227

[ref12] Bhuniya S.; Maiti S.; Kim E.-J.; Lee H.; Sessler J. L.; Hong K. S.; Kim J. S. An Activatable Theranostic for Targeted Cancer Therapy and Imaging. Angew. Chem., Int. Ed. 2014, 53 (17), 4469–4474. 10.1002/anie.201311133. 24644015

[ref13] Lee M. H.; Kim J. Y.; Han J. H.; Bhuniya S.; Sessler J. L.; Kang C.; Kim J. S. Direct Fluorescence Monitoring of the Delivery and Cellular Uptake of a Cancer-Targeted RGD Peptide-Appended Naphthalimide Theragnostic Prodrug. J. Am. Chem. Soc. 2012, 134 (30), 12668–12674. 10.1021/ja303998y. 22642558

[ref14] Reese A. E.; de Moliner F.; Mendive-Tapia L.; Benson S.; Kuru E.; Bridge T.; Richards J.; Rittichier J.; Kitamura T.; Sachdeva A.; McSorley H. J.; Vendrell M. Inserting “OFF-to-ON” BODIPY Tags into Cytokines: A Fluorogenic Interleukin IL-33 for Real-Time Imaging of Immune Cells. ACS Cent. Sci. 2024, 10 (1), 143–154. 10.1021/acscentsci.3c01125. 38292608 PMC10823590

[ref15] Liu Y.; Teng L.; Xu C.; Liu H.-W.; Xu S.; Guo H.; Yuan L.; Zhang X.-B. A “Double-Locked” and Enzyme-Activated Molecular Probe for Accurate Bioimaging and Hepatopathy Differentiation. Chem. Sci. 2019, 10 (47), 10931–10936. 10.1039/C9SC03628H. 32190249 PMC7066674

[ref16] Widen J. C.; Tholen M.; Yim J. J.; Antaris A.; Casey K. M.; Rogalla S.; Klaassen A.; Sorger J.; Bogyo M. AND-Gate Contrast Agents for Enhanced Fluorescence-Guided Surgery. Nat. Biomed. Eng. 2021, 5 (3), 264–277. 10.1038/s41551-020-00616-6. 32989286 PMC7969380

[ref17] Wang X.; Liew S. S.; Huang J.; Hu Y.; Wei X.; Pu K. Dual-Locked Enzyme-Activatable Bioorthogonal Fluorescence Turn-On Imaging of Senescent Cancer Cells. J. Am. Chem. Soc. 2024, 146 (32), 22689–22698. 10.1021/jacs.4c07286. 39101919

[ref18] Subiros-Funosas R.; Ho V. C. L.; Barth N. D.; Mendive-Tapia L.; Pappalardo M.; Barril X.; Ma R.; Zhang C.-B.; Qian B.-Z.; Sintes M.; Ghashghaei O.; Lavilla R.; Vendrell M. Fluorogenic Trp(redBODIPY) Cyclopeptide Targeting Keratin 1 for Imaging of Aggressive Carcinomas. Chem. Sci. 2020, 11 (5), 1368–1374. 10.1039/C9SC05558D. PMC8148049 34123261

[ref19] Scott J. I.; Mendive-Tapia L.; Gordon D.; Barth N. D.; Thompson E. J.; Cheng Z.; Taggart D.; Kitamura T.; Bravo-Blas A.; Roberts E. W.; et al. A Fluorogenic Probe for Granzyme B Enables In-Biopsy Evaluation and Screening of Response to Anticancer Immunotherapies. Nat. Commun. 2022, 13 (1), 2366 10.1038/s41467-022-29691-w. 35501326 PMC9061857

[ref20] Wang C.; Hong Y.; Dong L.; Cheng H.; Jin D.; Zhao R.; Yu Z.; Yuan Y. An AND-Gate Bioluminescent Probe for Precise Tumor Imaging. Chem. Sci. 2023, 14 (21), 5768–5773. 10.1039/D3SC00556A. 37265734 PMC10231332

[ref21] Barth N. D.; Van Dalen F. J.; Karmakar U.; Bertolini M.; Mendive-Tapia L.; Kitamura T.; Verdoes M.; Vendrell M. Enzyme-Activatable Chemokine Conjugates for In Vivo Targeting of Tumor-Associated Macrophages. Angew. Chem., Int. Ed. 2022, 61 (41), e202207508 10.1002/anie.202207508. PMC9826351 35993914

[ref22] Fernandez A.; Thompson E. J.; Pollard J. W.; Kitamura T.; Vendrell M. A Fluorescent Activatable AND-Gate Chemokine CCL2 Enables In Vivo Detection of Metastasis-Associated Macrophages. Angew. Chem., Int. Ed. 2019, 131 (47), 17050–17054. 10.1002/ange.201910955. PMC6900180 31535788

[ref23] Park S.-J.; Yeo H. C.; Kang N.-Y.; Kim H.; Lin J.; Ha H.-H.; Vendrell M.; Lee J.-S.; Chandran Y.; Lee D.-Y.; Yun S.-W.; Chang Y.-T. Mechanistic Elements and Critical Factors of Cellular Reprogramming Revealed by Stepwise Global Gene Expression Analyses. Stem Cell Res. 2014, 12 (3), 730–741. 10.1016/j.scr.2014.03.002. 24727632

[ref24] Cho H.; Hong N.-K.; Chang Y.-T. Novel Fluorescent Strategy for Discriminating T and B Lymphocytes Using Transport System. Pharmaceutics 2024, 16 (3), 424 10.3390/pharmaceutics16030424. 38543318 PMC10974409

[ref25] Cho H.; Kwon H.-Y.; Kim Y.; Kim K.; Lee E. J.; Kang N.-Y.; Chang Y.-T. Development of a Mature B Lymphocyte Probe through Gating-Oriented Live-Cell Distinction (GOLD) and Selective Imaging of Topical Spleen. JACS Au 2024, 4 (4), 1450–1457. 10.1021/jacsau.4c00001. 38665660 PMC11040558

[ref26] Mellanby R. J.; Scott J. I.; Mair I.; Fernandez A.; Saul L.; Arlt J.; Moral M.; Vendrell M. Tricarbocyanine N -Triazoles: The Scaffold-of-Choice for Long-Term Near-Infrared Imaging of Immune Cells in Vivo. Chem. Sci. 2018, 9 (36), 7261–7270. 10.1039/C8SC00900G. 30288247 PMC6148684

[ref27] Manukyan G.; Papajik T.; Mikulkova Z.; Urbanova R.; Kraiczova V. S.; Savara J.; Kudelka M.; Turcsanyi P.; Kriegova E. High CXCR3 on Leukemic Cells Distinguishes IgHV^mut^ from *IgH*_*V*_^unmut^ in Chronic Lymphocytic Leukemia: Evidence from CD5^high^ and CD5^low^ Clones. J. Immunol. Res. 2020, 2020, e7084268 10.1155/2020/7084268. PMC7322588 32802894

[ref28] Klier P. E. Z.; Gest A. M. M.; Martin J. G.; Roo R.; Navarro M. X.; Lesiak L.; Deal P. E.; Dadina N.; Tyson J.; Schepartz A.; et al. Bioorthogonal Fluorogenic Targeting of Voltage-Sensitive Fluorophores for Visualizing Membrane Potential Dynamics in Cellular Organelles. J. Am. Chem. Soc. 2022, 144 (27), 12138–12146. 10.1021/jacs.2c02664. 35776693 PMC9433336

[ref29] Salim A.; Werther P.; Hatzopoulos G. N.; Reymond L.; Wombacher R.; Gönczy P.; Johnsson K. Chemical Probe for Imaging of Polo-like Kinase 4 and Centrioles. JACS Au 2023, 3 (8), 2247–2256. 10.1021/jacsau.3c00271. 37654580 PMC10466336

[ref30] Agarwal P.; Beahm B. J.; Shieh P.; Bertozzi C. R. Systemic Fluorescence Imaging of Zebrafish Glycans with Bioorthogonal Chemistry. Angew. Chem., Int. Ed. 2015, 54 (39), 11504–11510. 10.1002/anie.201504249. PMC4694582 26230529

[ref31] Salic A.; Mitchison T. J. A Chemical Method for Fast and Sensitive Detection of DNA Synthesis in Vivo. Proc. Natl. Acad. Sci. U. S. A. 2008, 105 (7), 2415–2420. 10.1073/pnas.0712168105. 18272492 PMC2268151

[ref32] Nainar S.; Beasley S.; Fazio M.; Kubota M.; Dai N.; Corrêa Jr I. R.; Spitale R. C. Metabolic Incorporation of Azide Functionality into Cellular RNA. ChemBiochem 2016, 17 (22), 2149–2152. 10.1002/cbic.201600300. 27595557 PMC5115926

[ref33] Liang D.; Wu K.; Tei R.; Bumpus T. W.; Ye J.; Baskin J. M. A Real-Time, Click Chemistry Imaging Approach Reveals Stimulus-Specific Subcellular Locations of Phospholipase D Activity. Proc. Natl. Acad. Sci. U. S. A. 2019, 116 (31), 15453–15462. 10.1073/pnas.1903949116. 31311871 PMC6681737

[ref34] Alvarez-Castelao B.; Schanzenbächer C. T.; Hanus C.; Glock C.; Tom Dieck S.; Dörrbaum A. R.; Bartnik I.; Nassim-Assir B.; Ciirdaeva E.; Mueller A.; Dieterich D. C.; Tirrell D. A.; Langer J. D.; Schuman E. M. Cell-Type-Specific Metabolic Labeling of Nascent Proteomes in Vivo. Nat. Biotechnol. 2017, 35 (12), 1196–1201. 10.1038/nbt.4016. 29106408

[ref35] Yuet K. P.; Doma M. K.; Ngo J. T.; Sweredoski M. J.; Graham R. L. J.; Moradian A.; Hess S.; Schuman E. M.; Sternberg P. W.; Tirrell D. A. Cell-Specific Proteomic Analysis in *Caenorhabditis Elegans*. Proc. Natl. Acad. Sci. U. S. A. 2015, 112 (9), 2705–2710. 10.1073/pnas.1421567112. 25691744 PMC4352802

[ref36] Pinto-Pacheco B.; Carbery W. P.; Khan S.; Turner D. B.; Buccella D. Fluorescence Quenching Effects of Tetrazines and Their Diels–Alder Products: Mechanistic Insight Toward Fluorogenic Efficiency. Angew. Chem., Int. Ed. 2020, 59 (49), 22140–22149. 10.1002/anie.202008757. 33245600

[ref37] Aktalay A.; Lincoln R.; Heynck L.; Lima M. A. d. R. B. F.; Butkevich A. N.; Bossi M. L.; Hell S. W. Bioorthogonal Caging-Group-Free Photoactivatable Probes for Minimal-Linkage-Error Nanoscopy. ACS Cent. Sci. 2023, 9 (8), 1581–1590. 10.1021/acscentsci.3c00746. 37637742 PMC10450876

[ref38] Meimetis L. G.; Boros E.; Carlson J. C.; Ran C.; Caravan P.; Weissleder R. Bioorthogonal Fluorophore Linked DFO—Technology Enabling Facile Chelator Quantification and Multimodal Imaging of Antibodies. Bioconjugate Chem. 2016, 27 (1), 257–263. 10.1021/acs.bioconjchem.5b00630. PMC4858350 26684717

[ref39] Mendive-Tapia L.; Zhao C.; Akram A. R.; Preciado S.; Albericio F.; Lee M.; Serrels A.; Kielland N.; Read N. D.; Lavilla R.; et al. Spacer-Free BODIPY Fluorogens in Antimicrobial Peptides for Direct Imaging of Fungal Infection in Human Tissue. Nat. Commun. 2016, 7 (1), 10940 10.1038/ncomms10940. 26956772 PMC4786873

[ref40] Mendive-Tapia L.; Miret-Casals L.; Barth N. D.; Wang J.; de Bray A.; Beltramo M.; Robert V.; Ampe C.; Hodson D. J.; Madder A.; Vendrell M. Acid-Resistant BODIPY Amino Acids for Peptide-Based Fluorescence Imaging of GPR54 Receptors in Pancreatic Islets. Angew. Chem., Int. Ed. 2023, 62 (20), e202302688 10.1002/anie.202302688. PMC10947197 36917014

[ref41] Zhao C.; Fernandez A.; Avlonitis N.; Vande Velde G.; Bradley M.; Read N. D.; Vendrell M. Searching for the Optimal Fluorophore to Label Antimicrobial Peptides. ACS Comb. Sci. 2016, 18 (11), 689–696. 10.1021/acscombsci.6b00081. 27723293

[ref42] Chen L.; Li F.; Li Y.; Yang J.; Li Y.; He B. Red-Emitting Fluorogenic BODIPY-Tetrazine Probes for Biological Imaging. Chem. Commun. 2021, 58 (2), 298–301. 10.1039/D1CC05863K. 34889325

[ref43] Chi W.; Huang L.; Wang C.; Tan D.; Xu Z.; Liu X. A Unified Fluorescence Quenching Mechanism of Tetrazine-Based Fluorogenic Dyes: Energy Transfer to a Dark State. Mater. Chem. Front. 2021, 5 (18), 7012–7021. 10.1039/D1QM00852H.

[ref44] Vasco A. V.; Taylor R. J.; Méndez Y.; Bernardes G. J. L. On-Demand Thio-Succinimide Hydrolysis for the Assembly of Stable Protein–Protein Conjugates. J. Am. Chem. Soc. 2024, 146 (30), 20709–20719. 10.1021/jacs.4c03721. 39012647 PMC11295205

[ref45] Bernardim B.; Cal P. M. S. D.; Matos M. J.; Oliveira B. L.; Martínez-Sáez N.; Albuquerque I. S.; Perkins E.; Corzana F.; Burtoloso A. C. B.; Jiménez-Osés G.; et al. Stoichiometric and Irreversible Cysteine-Selective Protein Modification Using Carbonylacrylic Reagents. Nat. Commun. 2016, 7 (1), 13128 10.1038/ncomms13128. 27782215 PMC5095172

[ref46] Oliveira B. L.; Guo Z.; Bernardes G. J. L. Inverse Electron Demand Diels–Alder Reactions in Chemical Biology. Chem. Soc. Rev. 2017, 46 (16), 4895–4950. 10.1039/C7CS00184C. 28660957

[ref47] Rosenberg E. M.; Herrington J.; Rajasekaran D.; Murphy J. W.; Pantouris G.; Lolis E. J. The N-Terminal Length and Side-Chain Composition of CXCL13 Affect Crystallization, Structure and Functional Activity. Acta Crystallogr. Sect. Struct. Biol. 2020, 76 (10), 1033–1049. 10.1107/S2059798320011687. PMC7543660 33021505

[ref48] Proost P.; Schutyser E.; Menten P.; Struyf S.; Wuyts A.; Opdenakker G.; Detheux M.; Parmentier M.; Durinx C.; Lambeir A.-M.; Neyts J.; Liekens S.; Maudgal P. C.; Billiau A.; Van Damme J. Amino-Terminal Truncation of CXCR3 Agonists Impairs Receptor Signaling and Lymphocyte Chemotaxis, While Preserving Antiangiogenic Properties. Blood 2001, 98 (13), 3554–3561. 10.1182/blood.V98.13.3554. 11739156

[ref49] Lin P.; Zhou B.; Yao H.; Guo Y.-p Effect of Carboplatin Injection on Bcl-2 Protein Expression and Apoptosis Induction in Raji Cells. Eur. J. Histochem. 2020, 64 (3), 3134 10.4081/ejh.2020.3134. 32643899 PMC7366131

[ref50] Barroso R.; Muñoz L. M.; Barrondo S.; Vega B.; Holgado B. L.; Lucas P.; Baíllo A.; Sallés J.; Rodríguez-Frade J. M.; Mellado M. EBI2 Regulates CXCL13-Mediated Responses by Heterodimerization with CXCR5. FASEB J. 2012, 26 (12), 4841–4854. 10.1096/fj.12-208876. 22913878

[ref51] Mohamed A. N.; Al-Katib A. Establishment and Characterization of a Human Lymphoma Cell Line (WSU-NHL) with 14;18 Translocation. Leuk. Res. 1988, 12 (10), 833–843. 10.1016/0145-2126(88)90037-9. 3143865

[ref52] Versteegen R. M.; Rossin R.; ten Hoeve W.; Janssen H. M.; Robillard M. S. Click to release: Instantaneous doxorubicin elimination upon tetrazine ligation. Angew. Chem., Int. Ed. 2013, 52 (52), 14112–14116. 10.1002/anie.201305969. 24281986

