# Supplementary material for: Chemo-Click: Receptor-Controlled and Bioorthogonal Chemokine Ligation for Real-Time Imaging of Drug-Resistant Leukemic B Cells
Source: J Am Chem Soc. 2024 Oct 23;146(44):30565–72. doi: 10.1021/jacs.4c12035 (PMC11544607; doi:10.1021/jacs.4c12035)

Electronic Supporting Information

Chemo-Click: Receptor-Controlled and Bioorthogonal Chemokine Ligation for Real-time Imaging of Drug-Resistant Leukemic B Cells

Marco Bertolini, Lorena Mendive-Tapia, Utsa Karmakar, Marc Vendrell*

Centre for Inflammation Research, The University of Edinburgh, EH16 4UU Edinburgh, UK.

IRR Chemistry Hub, Institute for Regeneration and Repair, The University of Edinburgh, EH16 4UU Edinburgh, UK.

* Corresponding author e-mail address: marc.vendrell@ed.ac.uk.

Number of pages: 39

Number of figures: 22

Table of contents

1. Materials and Methods
2. Supplementary Figures
3. Supplementary Movies
4. NMR Spectra

1. Materials and Methods

Unless otherwise stated, all reactions were carried out under normal atmosphere in dried glassware. All chemicals were purchased from commercial sources and used as received. Compound BCN-PEG$_3$-Mal was purchased from Conju-Probe, LLC. Flash column chromatography was performed on a CombiFlash NextGen 100 provided with dual UV detection using prepacked normal phase (silica gel) columns. Thin layer chromatography was performed on pre-coated Merck silica gel 60 F_{254} plates, which were visualized under UV light at 254 nm and 365 nm. NMR spectra were recorded on 500 MHz or 600 MHz NMR spectrometers. Chemical shifts were reported in ppm (δ) as s (singlet), d (doublet), t (triplet), dd (doublet of doublet), m (multiplet), br s (broad singlet), and were referred to the solvent peak. HPLC-MS analysis was performed in an Agilent 1260 Infinity at 35°C using a Kinetex XB-C_{18} column (4.6 mm × 50 mm, 5 µm) with H_2O + 0.1% formic acid and ACN + 0.1% formic acid as mobile phases.

Chemical synthesis

3-bromo-5-(3-(1,3-dioxoisoindolin-2-yl)propoxy)benzaldehyde (1)

3-bromo-5-hydroxybenzaldehyde (400 mg, 2 mmol, 1 eq), N-(3-Bromopropyl)phthalimide (800 mg, 3 mmol, 1.5 eq) and K_2CO_3 (825 mg, 6 mmol, 3 eq) were weighed in a microwave glass vial and suspended in 8 mL of DMF. The reaction mixture was heated under microwave irradiation at 60°C for 1h. The resulting crude was evaporated by repeated azeotropization with toluene and dry-loaded onto silica gel. The crude was purified by flash column chromatography using a DCM/MeOH gradient. Compound **1** was isolated as an off-white powder (613 mg, 77%).

^{1}H NMR (500 MHz, $CDCl_3$): δ 9.85 (s, 1H), 7.84 (dd, J = 5.4, 3.1 Hz, 2H), 7.73 (dd, J = 5.5, 3.0 Hz, 2H), 7.55 (t, J = 1.5 Hz, 1H), 7.19 (dd, J = 2.5, 1.3 Hz, 1H), 7.15 (dd, J = 2.4, 1.8 Hz, 1H), 4.08 (t, J = 5.9 Hz, 2H), 3.92 (t, J = 6.7 Hz, 2H), 2.24 – 2.18 (m, 2H) ppm.

^{13}C NMR (126 MHz, $CDCl_3$): δ 190.6, 168.5, 160.0, 138.8, 134.2, 132.3, 125.9, 124.3, 123.6, 123.5, 113.0, 66.5, 36.9, 35.4, 31.8, 29.9, 28.2 ppm.

HRMS (ESI): m/z calculated for $C_{18}H_{15}BrNO_4$ $[M+H]^+$: 388.0179, found 388.0183.

2-(3-(3-bromo-5-(5,5-difluoro-1,3,7,9-tetramethyl-5H-dipyrrolo[1,2-c:2',1'-f][1,3,2]diazaborinin-10-yl)phenoxy)propyl)isoindoline-1,3-dione (2)

Compound **1** (305 mg, 0.8 mmol, 1 eq) was dissolved in dry DCM (33 mL) and the solution cooled down to 0ºC. To this solution, 2,4-dimethyl-1H-pyrrole (180 µL, 1.7 mmol, 2.2 eq) was added followed by two drops of TFA. The reaction was let stir at room temperature for 2 h. After this time, reaction was cooled down to 0ºC and DDQ (445 mg, 2.0 mmol, 2.5 eq) dissolved in 20 mL of DCM was added dropwise. After 2 h at room temperature, the reaction was cooled down to 0ºC and DIPEA (2 mL, 11.8 mmol, 15 eq), followed by $BF_3{\cdot}Et_2O$ (2 mL, 15.7 mmol, 20 eq), were added to the mixture. The reaction was stirred at room temperature for 3 h. Finally, the crude was diluted with DCM and filtered through cotton to remove precipitate. The filtrate was washed with water (×3), brine (×1) and dried over $MgSO_4$. Crude was dry loaded on silica gel and purification was performed by column chromatography in DCM. Compound **2** was obtained as a red film (300 mg, 63%).

^{1}H NMR (500 MHz, $CDCl_3$): δ 7.79 (dd, J = 5.5, 3.0 Hz, 2H), 7.68 (dd, J = 5.5, 3.0 Hz, 2H), 7.02 (t, J = 1.5 Hz, 1H), 6.98 (t, J = 1.7 Hz, 1H), 6.55 (dd, J = 2.4, 1.4 Hz, 1H), 5.99 (s, 2H), 4.03 (t, J = 5.8 Hz, 2H), 3.91 (t, J = 6.6 Hz, 2H), 2.55 (s, 6H), 2.22 – 2.16 (m, 2H), 1.50 (s, 6H) ppm.

^{13}C NMR (126 MHz, $CDCl_3$): δ 168.5, 160.2, 156.1, 143.1, 139.4, 137.6, 134.2, 132.2, 131.1, 123.5, 123.5, 123.4, 121.5, 118.4, 113.3, 66.6, 53.6, 35.6, 28.1, 14.8, 14.7 ppm.

HRMS (ESI): m/z calculated for $C_{30}H_{28}BBrN_3O_3F_2$ $[M+H]^+$: 606.1369, found 606.1365.

(3-(5,5-difluoro-1,3,7,9-tetramethyl-5H-dipyrrolo[1,2-c:2',1'-f][1,3,2]diazaborinin-10-yl)-5-(3-(1,3-dioxoisoindolin-2-yl)propoxy)phenyl)boronic acid (3)

Compound **2** (300 mg, 0.5 mmol, 1 eq), B_2pin_2 (150 mg, 0.6 mmol, 1.2 eq), potassium acetate (145 mg, 1.5 mmol, 3 eq) and $Pd(dppf)Cl_2$ (18 mg, 0.03 mmol, 0.05 eq.) were weighed and dissolved in dry 1,4-dioxane (3 mL) under N_2 atmosphere. The reaction was let stir at 80°C for 16 h. After this time, solvent was removed by rotary evaporation and the crude purified by flash column chromatography with a DCM/MeOH gradient. Solvent was removed and residue was

dissolved in ACN (40 mL) followed by addition of a 1% formic acid aqueous solution (20 mL). This reaction was let stir for 16 h at 50°C. After this time, the crude was purified by preparative reverse phase-HPLC (5-95%, 15 min) and lyophilized to obtain **3** as a red solid (140 mg, 71% yield over 2 steps).

^{1}H NMR (500 MHz, $CDCl_3$): δ 7.84 – 7.63 (m, 5H), 7.26 (m, 1H), 6.81 (m, 1H), 6.02 (s, 2H), 4.14 (dt, *J* = 26.5, 5.9 Hz, 2H), 3.96 (td, *J* = 6.6, 2.7 Hz, 2H), 2.58 (s, 6H), 2.26 (dt, *J* = 19.9, 6.1 Hz, 2H), 1.48 (s, 6H) ppm.

^{13}C NMR (126 MHz, $CDCl_3$): δ 168.4, 159.3, 155.7, 143.1, 141.0, 136.1, 135.9, 133.9, 132.1, 131.3, 126.9, 125.3, 123.3, 122.1, 121.3, 120.0, 118.5, 117.1, 66.2, 35.4, 29.7, 28.0, 14.6 ppm.

HRMS (ESI): m/z calculated for $C_{30}H_{30}B_2F_2N_3O_5$ $[M+H]^+$: 572.2334, found 572.2359.

2-(3-(3-(5,5-difluoro-1,3,7,9-tetramethyl-5H-dipyrrolo[1,2-c:2',1'-f][1,3,2]diazaborinin-10-yl)-5-(6-methyl-1,2,4,5-tetrazin-3-yl)phenoxy)propyl)isoindoline-1,3-dione (4)

Compound **3** (20 mg, 35 µmol, 1.9 eq), b-Tz (5.2 mg, 18 µmol, 1 eq), Ag_2O (12 mg, 46 µmol, 2.5 eq) and $Pd(dppf)Cl_2$ (5 mg, 3 µmol, 0.15 eq) were weighed in a Kimax tube, flushed with N_2 and suspended in 1 mL of dry DMF. The reaction was stirred at 60°C for 16 h. Subsequently, the crude was further diluted with DMF and filtered through a PTFE filter. This solution was purified by semipreparative reverse-phase HPLC (50%-95% ACN in H_2O over 15 min). Finally, the pooled fractions were neutralized by addition of an aqueous $NaHCO_3$ solution and extracted with DCM. After rotary evaporation, compound **4** was obtained as a red film (5 mg, 44%).

^{1}H NMR (500 MHz, $CDCl_3$): δ 8.15 (t, J = 1.5 Hz, 1H), 8.10 (dd, J = 2.5, 1.5 Hz, 1H), 7.78 (dd, J = 5.4, 3.0 Hz, 2H), 7.65 (dd, J = 5.4, 3.0 Hz, 2H), 6.83 (dd, J = 2.5, 1.4 Hz, 1H), 6.00 (s, 2H), 4.19 (t, J = 5.7 Hz, 2H), 3.96 (t, J = 6.6 Hz, 2H), 3.10 (s, 3H), 2.57 (s, 6H), 2.31 – 2.23 (m, 2H), 1.50 (s, 6H) ppm.

^{13}C NMR (126 MHz, $CDCl_3$): δ 168.4, 167.7, 163.5, 160.2, 155.9, 142.9, 139.9, 137.3, 134.1, 134.0, 132.1, 131.1, 123.3, 121.4, 120.1, 119.0, 113.4, 66.5, 35.5, 29.7, 28.0, 21.2, 14.7, 14.6 ppm.

HRMS (ESI): m/z calculated for $C_{33}H_{31}BF_2N_7O_3$ [M + H]$^+$: 622.2544, found 622.2570.

N-(3-(3-(5,5-difluoro-1,3,7,9-tetramethyl-5H-dipyrrolo[1,2-c:2',1'-f][1,3,2]diazaborinin-10-yl)-5-(6-methyl-1,2,4,5-tetrazin-3-yl)phenoxy)propyl)-3-(2-(2-(3-(2,5-dioxo-2,5-dihydro-1H-pyrrol-1-yl)propanamido)ethoxy)ethoxy)propanamide (5)

Compound **4** (20 mg, 30 µmol, 1 eq) was dissoved in MeOH (5 mL) and $MeNH_2$ (33% wt in ethanol, 110 µL, 960 µmol, 30 eq) was added. Reaction was heated to 50°C and stirred for 16 h. The reaction was diluted in water and extracted with EtOAc (×3). The organic phase was further washed with water (×2) and aqueous $NaHCO_3$ (×2) before being dried with $MgSO_4$ and the solvent removed. In the same flask, maleimide-PEG2-succinimidyl ester (19 mg, 44 µmol, 1.5 eq) was weighed and both compounds were dissolved in dry DMF (3 mL). To this solution, DIPEA (150 µL, 880 µmol, 30 eq) was added and the reaction was stirred at room temperature for 1 h. After this time, the crude was purified by semi-preparative reverse phase HPLC (5-95% ACN in H_2O over 17 min). After lyophilization, compound **5** was obtained as a red solid (9 mg, 40%).

HRMS (ESI): m/z calculated for $C_{39}H_{46}B_1F_2N_9O_7Na_1$ [M+Na]$^+$: 824.3473, found 824.3474.

HPLC-MS: retention time 5.3 min, 99% purity.

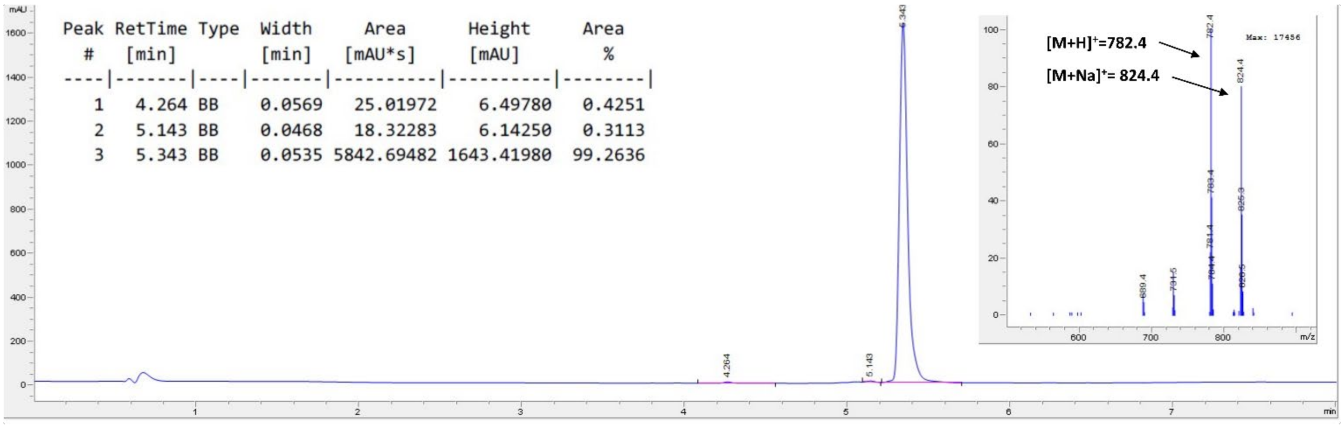

Peak #	RetTime [min]	Type	Width [min]	Area [mAU*s]	Height [mAU]	Area %
1	4.264	BB	0.0569	25.01972	6.49780	0.4251
2	5.143	BB	0.0468	18.32283	6.14250	0.3113
3	5.343	BB	0.0535	5842.69482	1643.41980	99.2636

Kinetic rate constant determination. Rate constant k was measured for compound **4** under pseudo-first-order conditions with an excess of BCN in a final mixture of 25% DMSO in water. Reaction was monitored by measuring the exponential fluorescence increase at 520 nm (λ_{ex}: 490 nm) using a Varioskan Lux spectrophotometer equipped with an automatic dispenser. Briefly, a stock solution of compound **4** in DMSO (5 mM) was diluted in water and transferred in a 96-well plate. Next, a stock of BCN (10 mM) in DMSO was loaded onto the dispenser syringe. All measurements were conducted at 25ºC. After starting the recording, different volumes of BCN stock solution were dispensed in the wells containing compound **4** followed by a 2 sec shaking step. Addition of BCN to the wells resulted in a final concentration of 10 µM for compound **4** and 0.7-1 mM for BCN. Each measurement was perfomed in triplicate. Baseline fluorescence was subtracted from data points and these were fitted using a non linear one-phase association equation using GraphPad Prism. The mean of the observed rates k' was plotted against the concentration of BCN. The rate constant k was obtained from the slope of the plot.

Cell culture. Raji and MCF-7 cells were obtained from American Type Culture Collection (ATCC). WSU-NHL were obtained from The German Cancer Research Center (DKFZ). All cells were cultured in RPMI 1640 (Gibco) containing 2 mM glutamine (Gibco), and supplemented with 10% fetal bovine serum (FBS, Gibco) and antibiotics (100 U mL^{-1} penicillin, 100 µg mL^{-1} streptomycin, Gibco). Cells were maintained in a humidified atmosphere at 37°C with 5% CO_2.

Cytotoxicity assays. MCF-7 cells (5,000 cells/well) were seeded into a 96-well plate and incubated in 100 µL complete medium overnight at 37°C. The next day, BCN and compound **4** were each dissolved in DMSO to a concentration of 20 mM. These solutions were then mixed in a 1:1 ratio to create a 10 mM stock solution of BCN-clicked compound **4**, which was incubated at room temperature for 30 min. These stock solutions were diluted in complete medium to a final concentration of 100 µM and added to the cells. The cells were incubated overnight at 37°C.

The following day, a 0.1% resazurin solution (Sigma-Aldrich) in water was prepared, and 10 µL of this solution was added to each well. The plate was incubated at 37°C for approximately 12 hours. Fluorescence was then measured using a Varioskan Lux spectrophotometer with excitation at 560 nm and emission at 590 nm. Untreated cells were used as a control to normalize the data to 100% viability. The experiment was performed in triplicate, and the data were analyzed using GraphPad Prism.

Chemokine conjugates production, purification and characterization. The human chemokines hCXCL10-Cys and hCXCL13-Cys were supplied by Almac. 20 µL of compound **5** (1.5 mM) or BCN-PEG$_3$-maleimide (10 mM) in dry DMSO were added to a solution of hCXCL13-Cys (100 µg, 0.009 µmol) or hCXCL10-Cys (100 µg, 0.011 µmol) previously dissolved in phosphate buffer (180 µL, 10 mM, pH 7). The mixtures were allowed to stir at room temperature for 1 h. Precipitants were removed using Costar Spin-X Centrifuge Tube Filters (0.45 µm, Corning) according to manufacturer's indications. Next, labeling agent excess was removed by ultracentrifugation with Amicon Filters (Merck, 0.5 mL, 3 kDa MWCO). Briefly, the crude reaction was diluted to 500 µL using phosphate buffer, loaded onto the filtration unit and centrifuged at 14,000 *g* for 20 min at 4°C. This procedure was repeated 5 times to ensure complete removal of labeling agent excess. Following purification, the concentrations of the chemokine conjugates were determined using the Coomassie Plus Bradford Assay Kit (Thermo Fisher) in accordance with the manufacturer's guidelines. SDS-PAGE was performed with NuPAGE 4-12% Bis-Tris Gels (Invitrogen). Precast gels were loaded with 1.5 µg protein in non-reducing Laemmli SDS sample buffer to run the gel for 45 min at 200 V in NuPAGE™ MOPS SDS Running Buffer (Invitrogen). For Coomassie-based protein visualization, the gel was subjected to a 3 h incubation in Coomassie staining solution, and then destained in milliQ H_2O. In-gel fluorescence was acquired on a ChemiDoc Imaging System (Bio-Rad) at 500 nm. Labeling was confirmed by

electrospray ionization mass spectrometry coupled with liquid chromatography. After purification, the chemokine solution was snap-frozen in liquid nitrogen and stored at -80°C.

Confocal microscopy experiments. hCXCL13-5 and hCXCL10-5 in Raji and WSU-NHL cells. Raji and WSU-NHL cells (3 x 10^5 cells in 100 µL of DPBS) were treated with 0.5 µM of **hCXCL13-5** and 0.5 µM of **hCXCL10-5**, respectively. The cells were incubated for 15 min at 37°C. Following incubation, a washing step was performed by centrifugation (350 x *g*, 5 min, r.t.) and addition of 100 µL DPBS. After another round of centrifugation, cells were resuspended in 100 µL DPBS containing CellMask Deep Red (1:2000 dilution) and 50 µM BCN, and kept in the dark for 15 min at 4°C before being imaged.

Dual chemokine ligation. For dual chemokine imaging in Raji and WSU-NHL, cells (3 x 10^5 cells in 100 µL DPBS) were incubated with 1 µM **hCXCL10-BCN** for 15 min at 37°C. Cells were spun down (350 x *g*, 5 min, r.t.) and then resuspended in 100 µL of DPBS containing Lysotracker Red (1:1000, Invitrogen) and CellMask Deep Red (1:2000 dilution). Cells were incubated in the dark for 15 min at 4°C before being transferred to an Ibidi 18-well chamber slide pre-coated with poly-D-lysine. Cells were allowed to settle for 10 min in the microscope thermostatted chamber set at 37°C. After recording was started, 1 µL **hCXCL13-5** (440 nM) was added to the well and fluorescence recorded for approximately 10-15 min.

Dual chemokine ligation in Raji and WSU-NHL co-culture. Raji and WSU-NHL cells were harvested and resuspended separately in DPBS at 2.5 x 10^6 cells mL^{-1}. DRAQ5 (5 µM) was added to the Raji cell suspension, followed by incubation at r.t. for 10 min. After incubation, cells were washed and resuspended in DPBS. Equal volumes (50 µL) of each cell suspension (1.25 x 10^5 cells, 1:1 ratio) were then combined and incubated with **hCXCL10-BCN** (1 µM) for 15 min at 37°C. The co-cultured cells were pelleted (350 x *g*, 5 min, r.t.), resuspended in 100 µL **hCXCL13-5** (440 nM) in DPBS, and incubated for 10 min at r.t. before analysis. All imaging experiments were conducted on a FALCON SP8 confocal microscope equipped with a HC PL

APO CS2 63x objective and a thermostatted chamber set at 37°C. Image analysis was carried out using LAS X and ImageJ software.

Flow cytometry. Titration with anti-CXCR5 and anti-CXCR3 antibodies. To validate expression of CXCR5 and CXCR3 chemokine receptors in tested cell lines a titration experiment with commercial antibodies was performed. Briefly, Raji and WSU-NHL cells (5×10^5 per condition) were incubated with serial dilutions of anti-hCXCR3-PE antibody (clone CEW33D, eBioscience) and anti-hCXCR5-AF647 (clone J252D4, BioLegend) in DPBS for 30 min at 4°C. Next, cells were washed with DPBS, resuspended in FACS buffer.

hCXCL13-5 concentration optimization. Raji cells were harvested and 5×10^5 cells per condition were used. Cells were initially incubated with increasing concentrations of **hCXCL13-5** (from 45 nM to 440 nM) for 30 min at 37°C. Subsequently, cells were washed and resuspended in 200 µL of BCN (100 uM) in DPBS.

Dual chemokine activation. Raji and WSU-NHL cells were harvested and resuspended at 5×10^6 cells mL^{-1} in DPBS, 100 µL/well were transferred into Eppendorf tubes. To this cell suspension, **hCXCL13-5** at a final concentration of 220 nM was added and cells were incubated at 37°C for 30 min. After treatment, cells were centrifuged (350 x *g*, 5 min, r.t.), resuspended in DPBS (100 µL) and incubated with 290 nM **hCXCL10-BCN** at 37°C for 30 min. Next, cells were centrifuged (300 x *g*, 5 min, r.t.), resuspended in 200 µL FACS buffer ready for analysis. A minimum of 10,000 events per condition were recorded.

Antibody blocking assay. WSU-NHL cells were harvested and resuspended in DPBS at 5×10^6 cells mL^{-1}. For each condition, 100 µL of this suspension were used. **hCXCL13-5** was added to a final concentration of 500 ng mL^{-1}, with or without neutralizing anti-hCXCR3 antibody (20 µg mL^{-1}, clone MAB160), and cells were incubated at 37°C for 30 min. After incubation, cells were centrifuged (350 x *g*, 5 min, r.t.), resuspended in 100 µL of DPBS, and incubated with 500 ng mL^{-1} of **hCXCL10-BCN** at 37°C for 30 min, again in the presence or absence of neutralizing anti-hCXCR3 antibody (20 µg mL^{-1}). Cells were then centrifuged (350 x *g*, 5 min, r.t.),

resuspended in 200 µL FACS buffer. All above experiments were conducted on an Attune Cytpix flow cytometer (Thermo Fisher) and data analyzed using FlowJo software.

Kinetic flow assays. WSU-NHL cells were harvested, centrifuged at 350 x *g* for 5 min and the cell pellets were resuspended in DPBS. Chemokine **hCXCL13-5** was prepared at a concentration of 5 µg mL^{-1} (470 nM). For staining, 0.5 million cells were collected and resuspended in 50 µL of DPBS. Then, 50 µL 2X **hCXCL13-5** solution was added to the cells to achieve a final concentration of 2.5 µg mL^{-1} (235 nM) in FACS buffer (PBS with 1% BSA). The cells were incubated at 37°C for 30 min. The activating chemokine **hCXCL10-BCN** was prepared at a final concentration of 5 µg mL^{-1} (280 nM) in FACS buffer. After 30 min incubation with **hCXCL13-5**, the cells were washed to remove excess chemokine, and then resuspended in 100 µL of FACS buffer. The flow cytometer was set up with appropriate gates and voltages. Cell suspension acquisition was started at a low setting (5 µL mL^{-1}). After a few minutes of baseline acquisition, the **hCXCL10-BCN** solution was added to the wells containing the cells. Acquisition was monitored for as long as possible to capture the dynamic response. Data were acquired on a NovoCyte (Agilent) flow cytometer and plotted using FCS Express.

Transwell migration assay. WSU-NHL cells were harvested and resuspended in RPMI to a concentration of 5 x 10^6 cells mL^{-1}. 100 µL of the cell suspension were added into the wells of a HTS Transwell®-96 Permeable Support with 5.0 µm Pore Polycarbonate Membrane (Corning). The support was placed into a 96-well plate with 200 µL RPMI or RPMI containing 313 ng mL^{-1}, 625 ng mL^{-1}, 1,250 ng mL^{-1} or 2,500 ng mL^{-1} of chemokine conjugates or chemokine thiol (control). Cells were allowed to migrate for 12 h in a humidified atmosphere at 37°C with 5% CO_2. The plate was gently centrifuged (200 x *g*, 5 min, r.t.) and the support removed. Migrated cells were supplemented with 10 µL 1% resazurinand incubated at 37°C for 1 day. The next day, the fluorescent emission ($\lambda_{exc.}$: 560 nm, $\lambda_{em.}$: 590 nm) was recorded for all wells using a VarioSkan Lux spectrophotometer. Each condition was analysed in triplicate. Relative migration

was calculated as ratio between intensity measured in stimulated cells and the intensity of cells incubated with RPMI only (basal migration). Data analysis was performed using Graph Pad Prism.

qPCR analysis. RNA was extracted from Raji and WSU-NHL cells using a QIAGEN RNA extraction kit (74104) and reverse transcribed with Vilo Superscript (ThermoFisher, 11754250). Preparation of cDNAs and analysis by qPCR were performed according to conventional protocols (Bombail et al. *J. Clin. Endocrinol. Metabol.*, 2010, 95, E224–E228). CXCR3 and CXCR5 gene expression was measured using primer/probe assays from ThermoFisher Scientific (Hs01847760_s1 and Hs00540548_s1, respectively) and standard run Taqman gene expression mastermix (Life Technologies). Samples were quantified by the comparative ΔΔCt method with cyclophilin A as an internal control.

2. Supplementary Figures

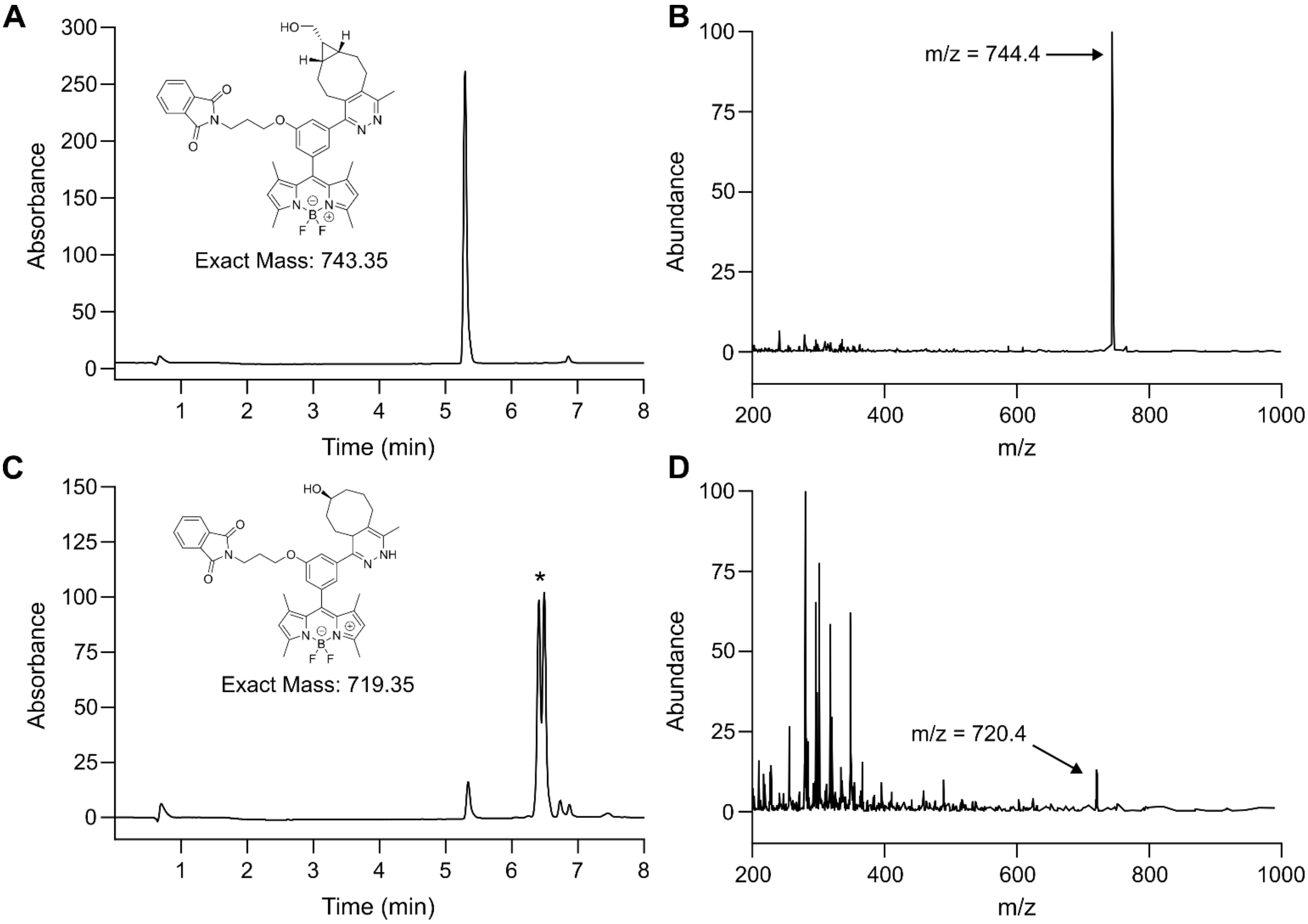

Figure S1. HPLC analysis of the click reaction between compound **4** and excess BCN or TCO. A) HPLC traces of the reaction between **4** and BCN (100 eq) and B) mass spectrum of the product. C) HPLC trace of the reaction between **4** and TCO (100 eq) and D) mass spectrum of the products. Both reactions were analysed after incubation at r.t. for 30 min. UV detection: 500 nm. * = two isomers are formed and can be observed as twin peaks.

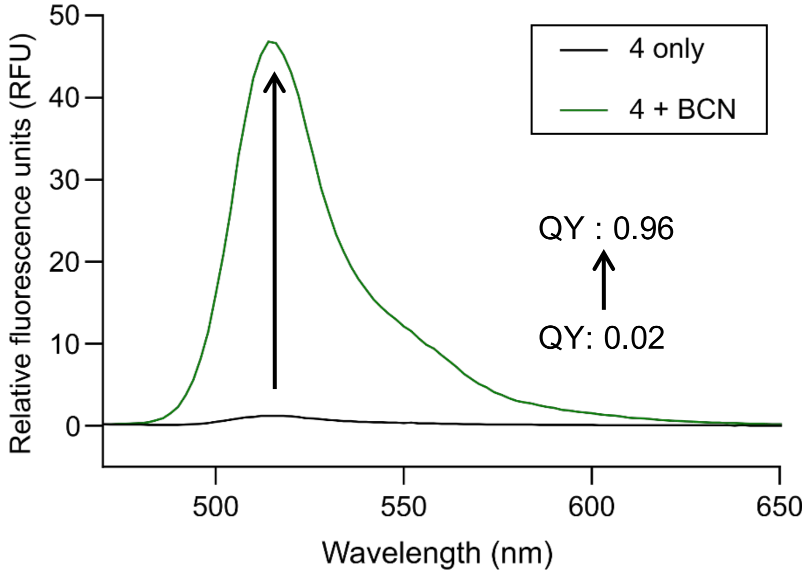

Figure S2. Representative fluorescence spectra of compound **4** (25 μM) alone (black) and after reaction with BCN (100 eq, green) in DMSO. λ_{exc}: 450 nm (n=3).

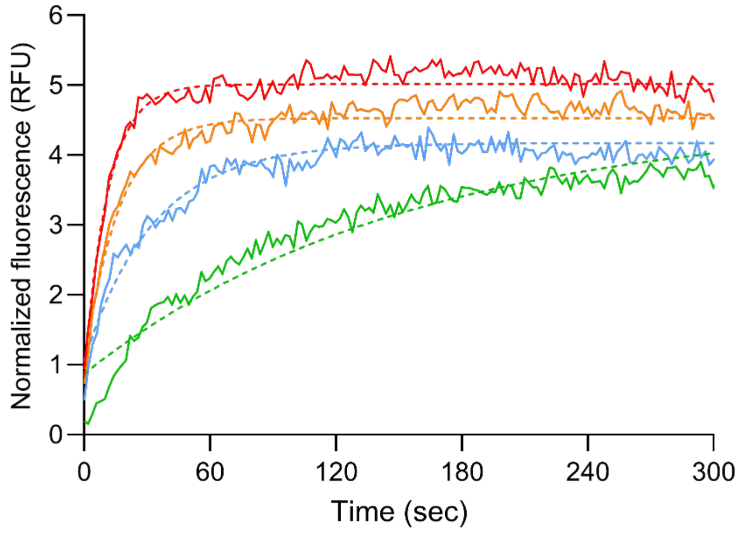

Figure S3. Time-lapse fluorescence emission of compound **4** using plate reader equipped with dispensers. Compound **4** (5 μM) was reacted with BCN in 25% DMSO/water at 25°C. Data presented as average (solid lines) of 3 independent experiments and fitted with a one-phase association function (dashed lines). Equivalents of BCN used in the experiments: 70 (green), 80 (blue), 90 (orange), 100 (red).

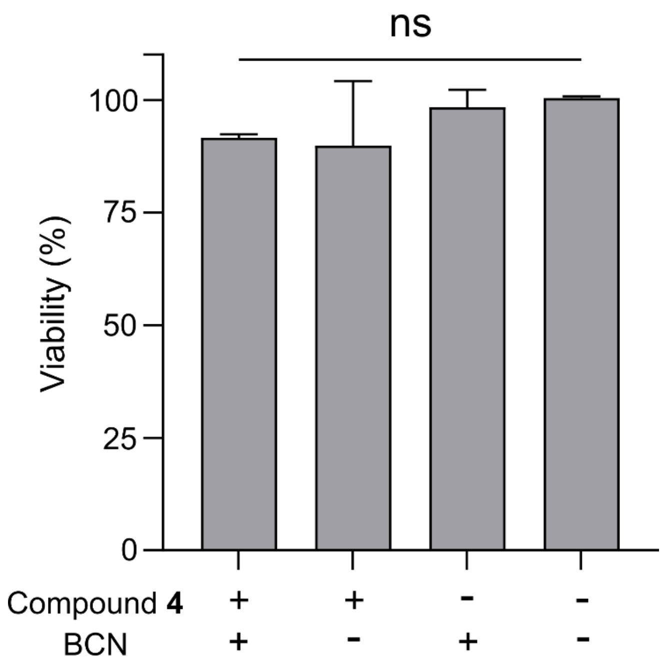

Figure S4. Viability assays of MCF-7 cells incubated with compound **4** (100 μM), BCN (100 μM) or **BODIPY 4-BCN** (100 μM) for 18 h at 37°C. Data presented as means±SD (n=3). P values determined by one-way ANOVA; ns for $p > 0.05$.

A

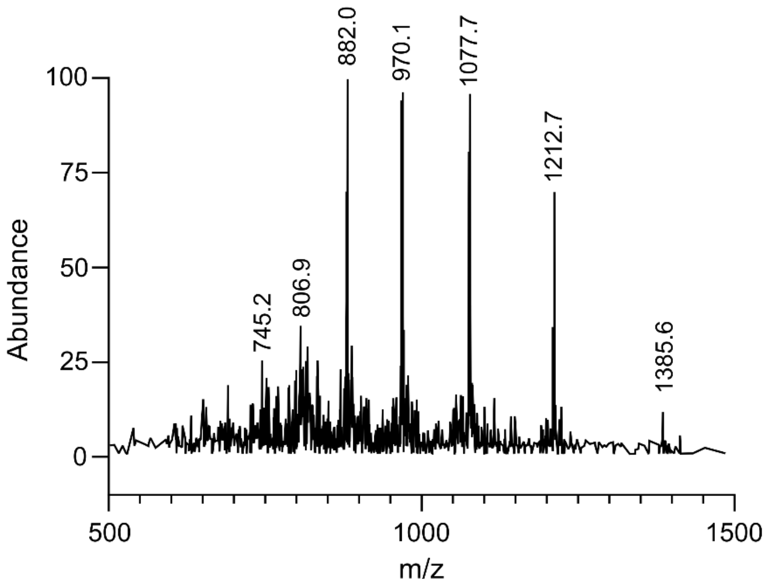

hCXCL10-SH mass (Da)	8890.1
Compound 5 mass (Da)	801.4

hCXCL10-5	m/z
[M]$^{+}$	9691.8
[M + 7H]$^{7+}$	1385.5
[M + 8H]$^{8+}$	1212.5
[M + 9H]$^{9+}$	1077.9
[M + 10H]$^{10+}$	970.2
[M + 11H]$^{11+}$	882.1
[M + 12H]$^{12+}$	808.6
[M + 13H]$^{13+}$	746.5

B

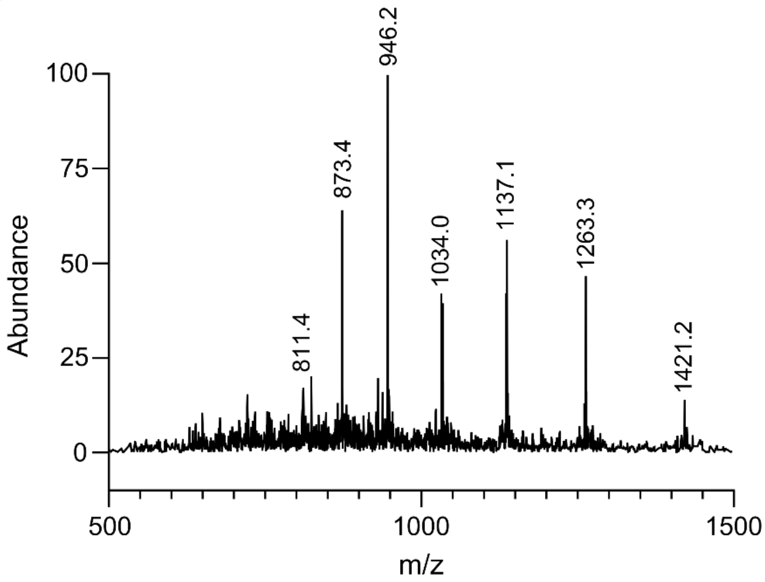

hCXCL13-SH mass (Da)	10561.9
Compound 5 mass (Da)	801.4

hCXCL13-5	m/z
[M]$^{+}$	11363.5
[M + 8H]$^{8+}$	1421.4
[M + 9H]$^{9+}$	1263.6
[M + 10H]$^{10+}$	1137.4
[M + 11H]$^{11+}$	1034.0
[M + 12H]$^{12+}$	948.0
[M + 13H]$^{13+}$	875.1
[M + 14H]$^{14+}$	812.7

Figure S5. Mass spectrometry analysis of **hCXCL10-5** (A) and **hCXCL13-5** (B), displaying mass spectra with major m/z ions detected (left) and calculated m/z ions (right).

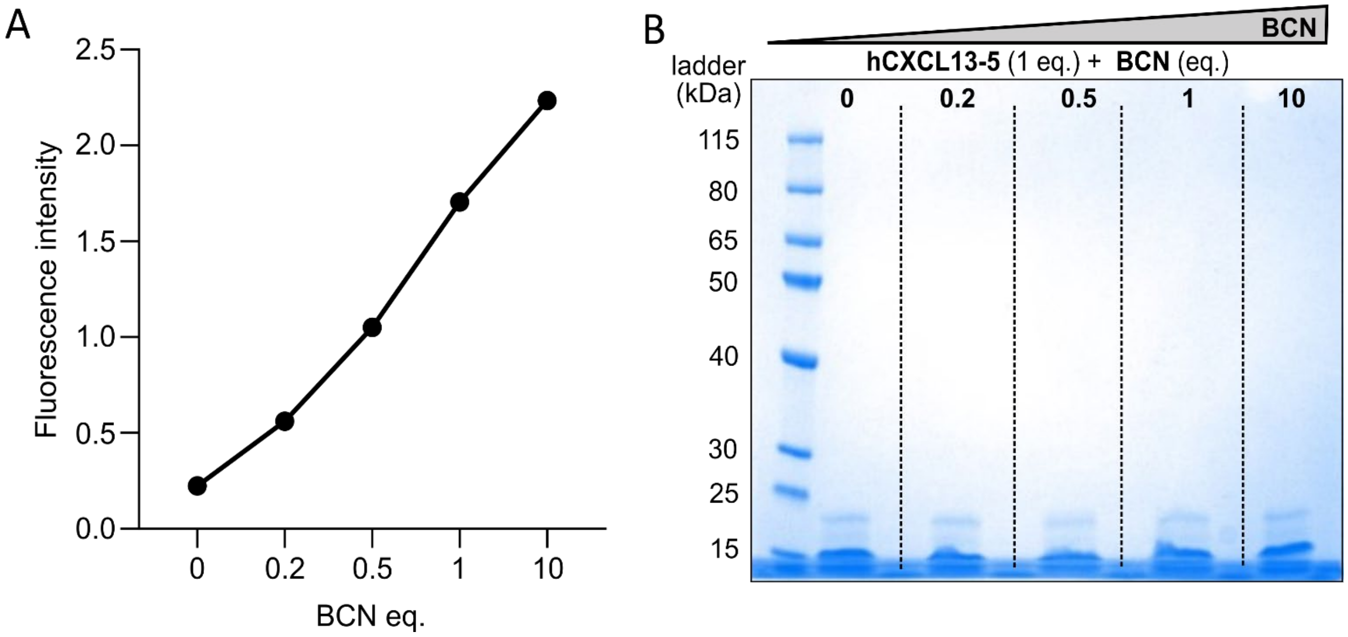

Figure S6. Representative fluorescence intensity quantification (A) and Coomassie stained SDS-PAGE (B) of crude mixtures containing **hCXCL13-5** (1 eq. = 1.5 μg) and increasing amounts of BCN (0-10 eq.).

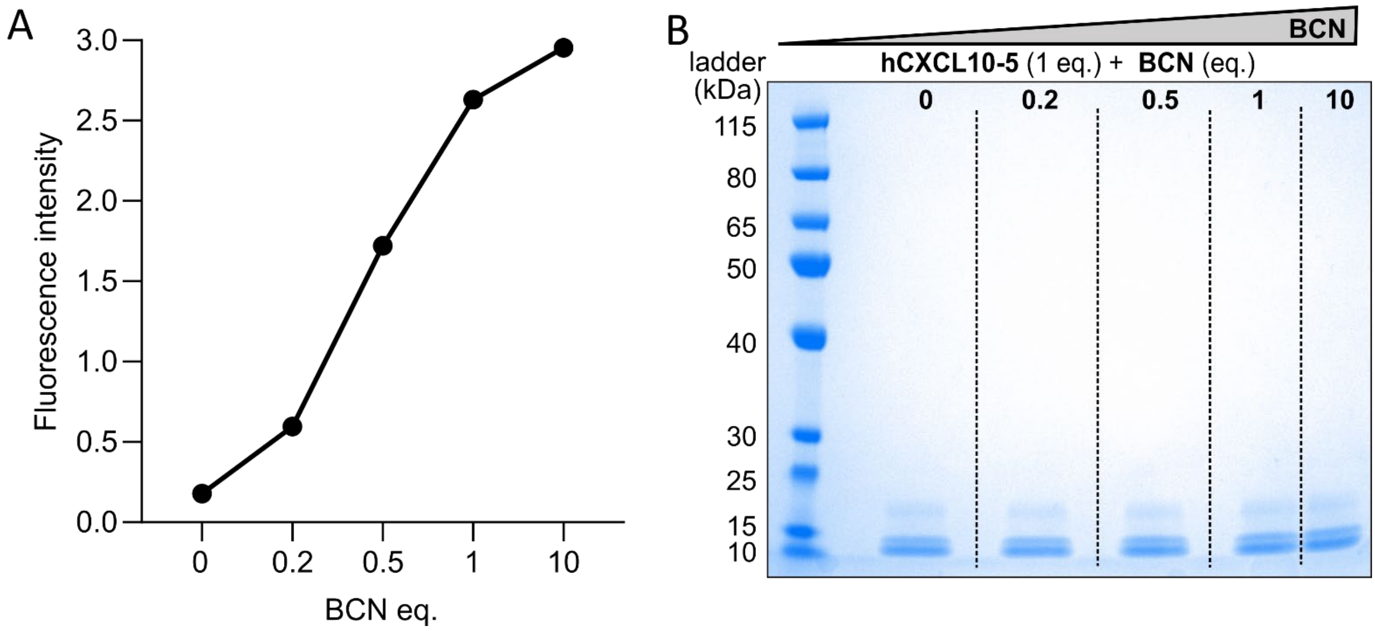

Figure S7. Representative fluorescence intensity quantification (A) and Coomassie stained SDS-PAGE gel (B) of crude mixtures containing **hCXCL10-5** (1 eq. = 1.5 µg) and increasing amounts of BCN (0-10 eq.).

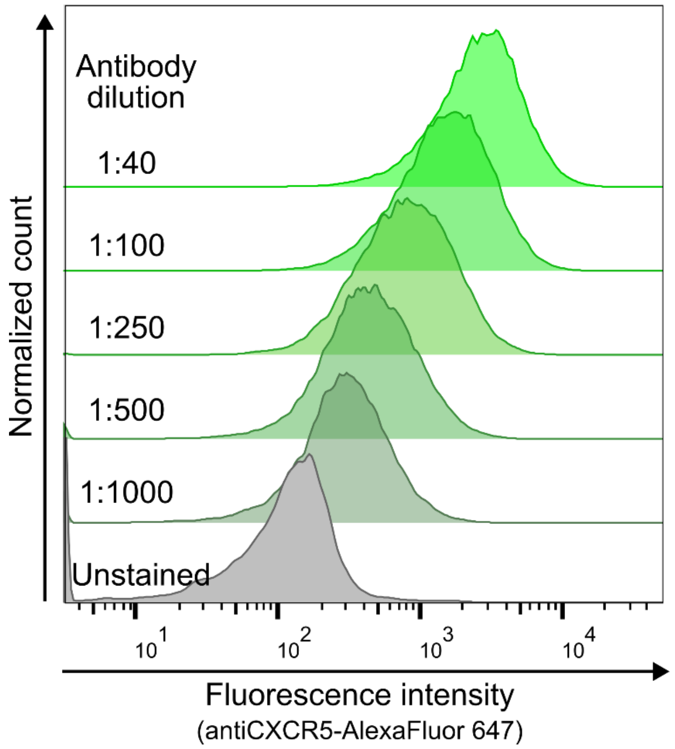

Figure S8. Fluorescence histograms of Raji cells after titration with commercial anti-hCXCR5 antibody (100 µg mL^{-1}) after flow cytometry analysis.

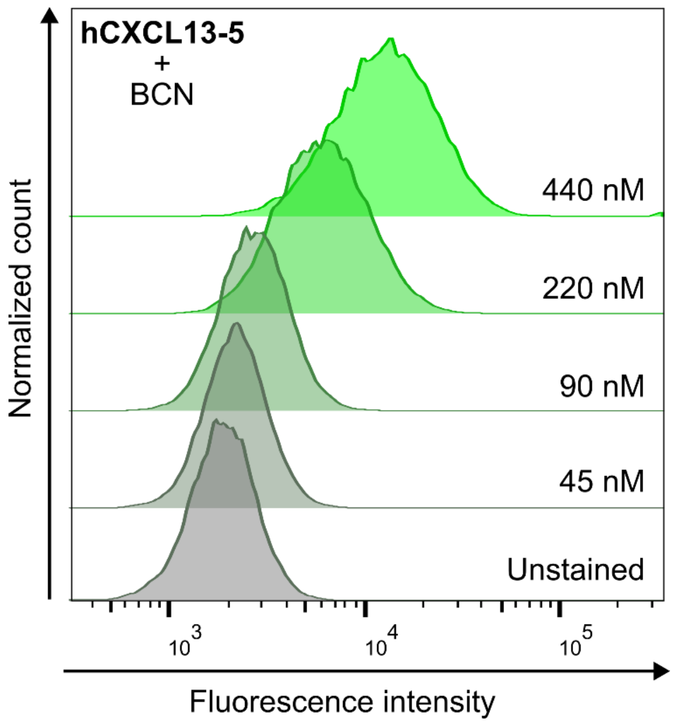

Figure S9. Fluorescence flow cytometry histograms of Raji cells following titration with increasing concentrations of **hCXCL13-5** and subsequent activation with BCN (100 µM).

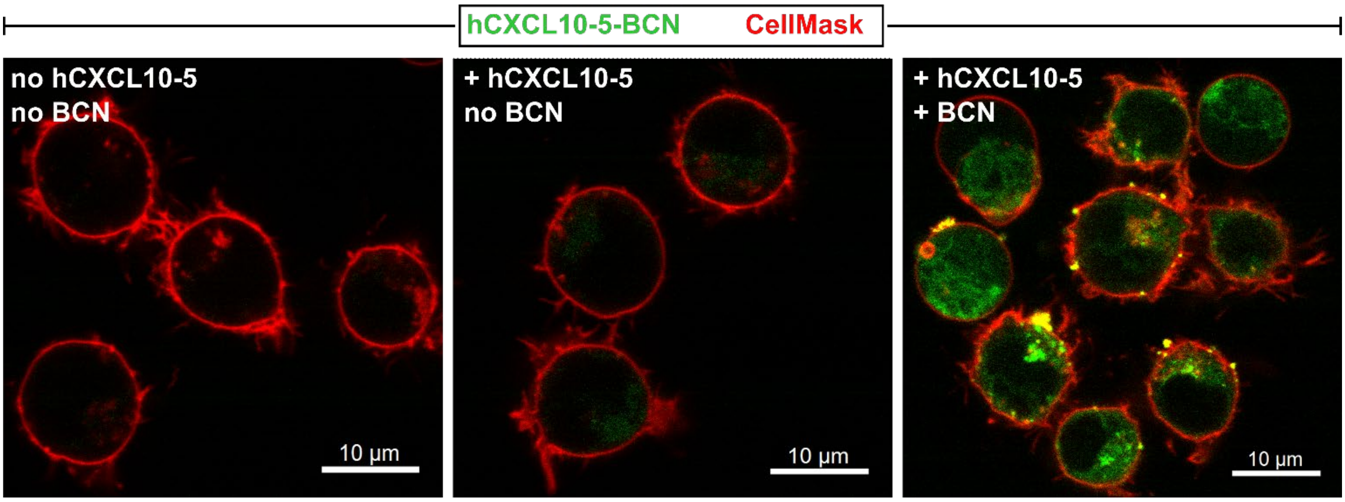

Figure S10. Representative confocal microscopy images (from 2 independent experiments) of WSU-NHL cells incubated with chemokine **hCXCL10-5** (500 nM, green) in the presence or absence of BCN (50 µM) and CellMask Deep Red (1:2000 dilution, red). Excitation wavelengths: 488 nm (for **hCXCL10-5**), 660 nm (for CellMask Deep Red). Scale bar: 10 µm.

A

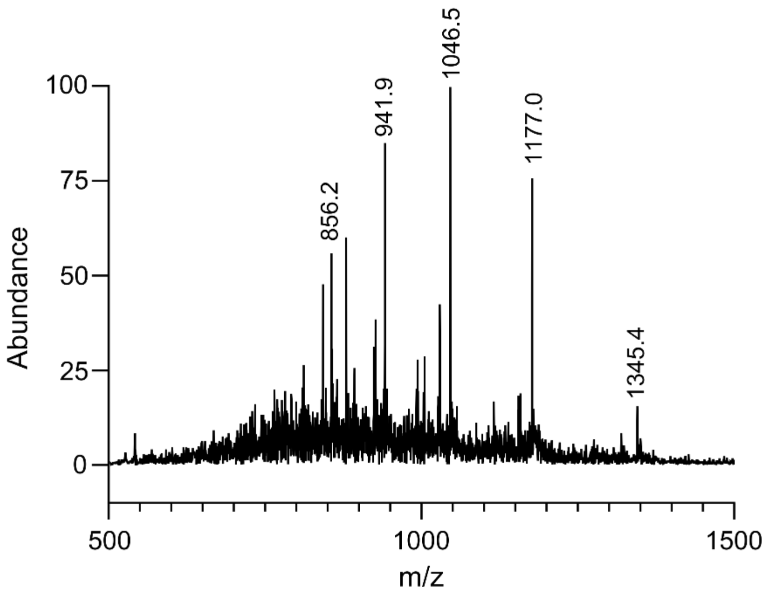

hCXCL10-SH mass (Da)	8890.1
Maleimide-PEG$_3$-BCN mass (Da)	519.6

hCXCL10-BCN	m/z
$[M]^+$	9409.7
$[M + 7H]^{7+}$	1345.2
$[M + 8H]^{8+}$	1177.2
$[M + 9H]^{9+}$	1046.5
$[M + 10H]^{10+}$	942.0
$[M + 11H]^{11+}$	856.4

B

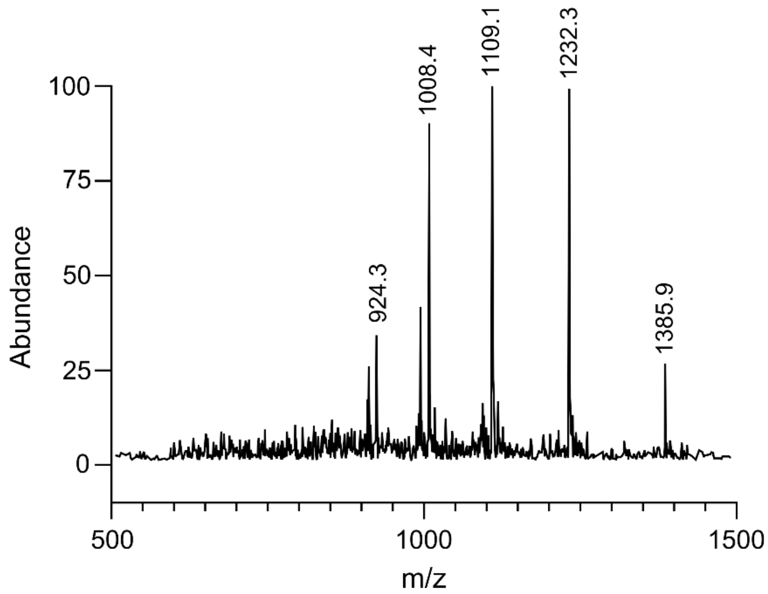

hCXCL13-SH mass (Da)	10561.9
Maleimide-PEG$_3$-BCN mass (Da)	519.6

hCXCL13-BCN	m/z
$[M]^+$	11081.5
$[M + 8H]^{8+}$	1386.2
$[M + 9H]^{9+}$	1232.3
$[M + 10H]^{10+}$	1109.1
$[M + 11H]^{11+}$	1008.4
$[M + 12H]^{12+}$	924.5

Figure S11. Mass spectrometry analysis of **hCXCL10-BCN** (A) and **hCXCL13-BCN** (B), displaying mass spectra with major m/z ions detected (left) and calculated m/z ions (right).

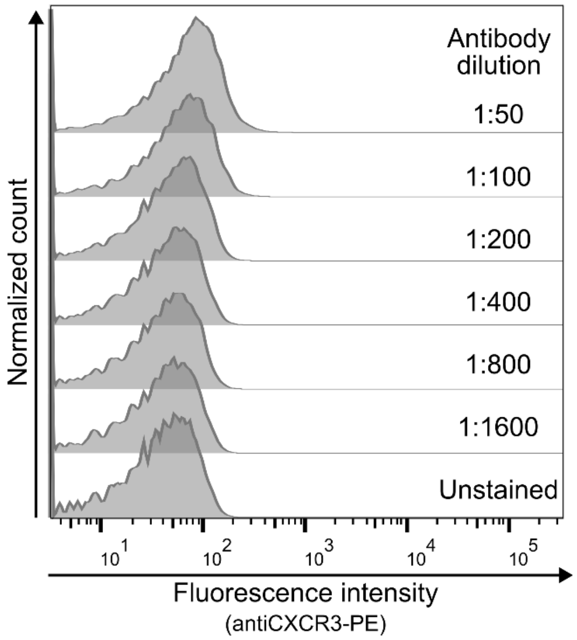

Figure S12. Fluorescence histograms of Raji cells after titration with commercial anti-hCXCR3 antibody (100 µg mL^{-1}) after flow cytometry analysis.

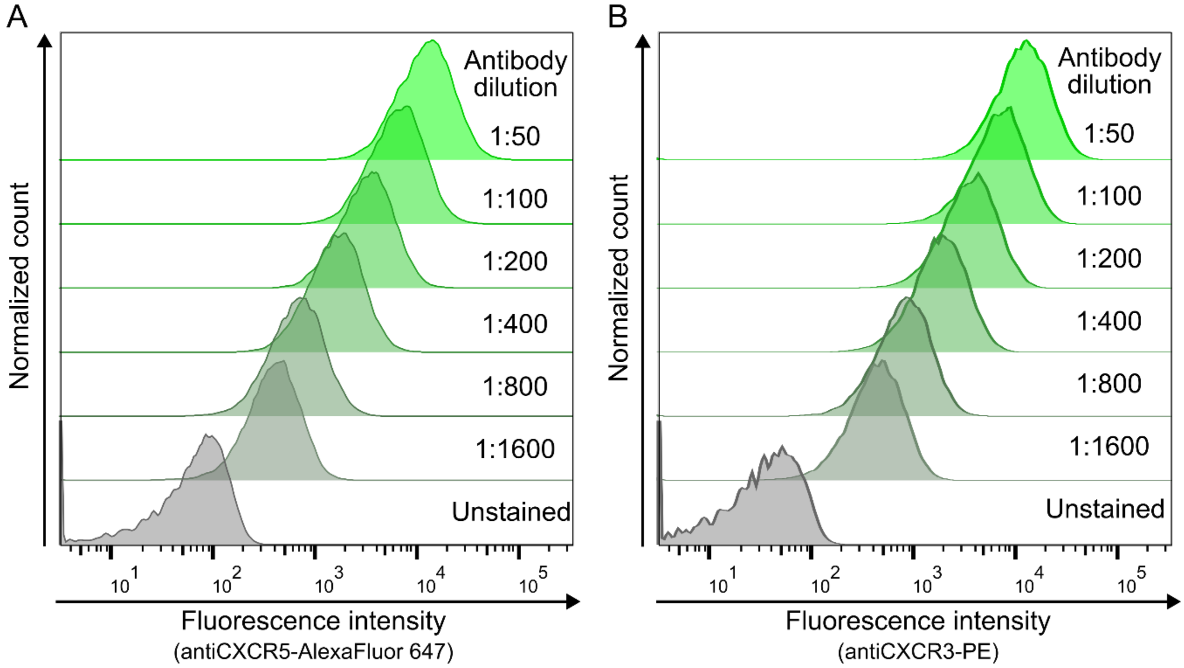

Figure S13. Fluorescence histograms of WSU-NHL cells after titration with commercial anti-hCXCR5 (100 µg mL^{-1}) and anti-hCXCR3 (100 µg mL^{-1}) antibodies using flow cytometry analysis.

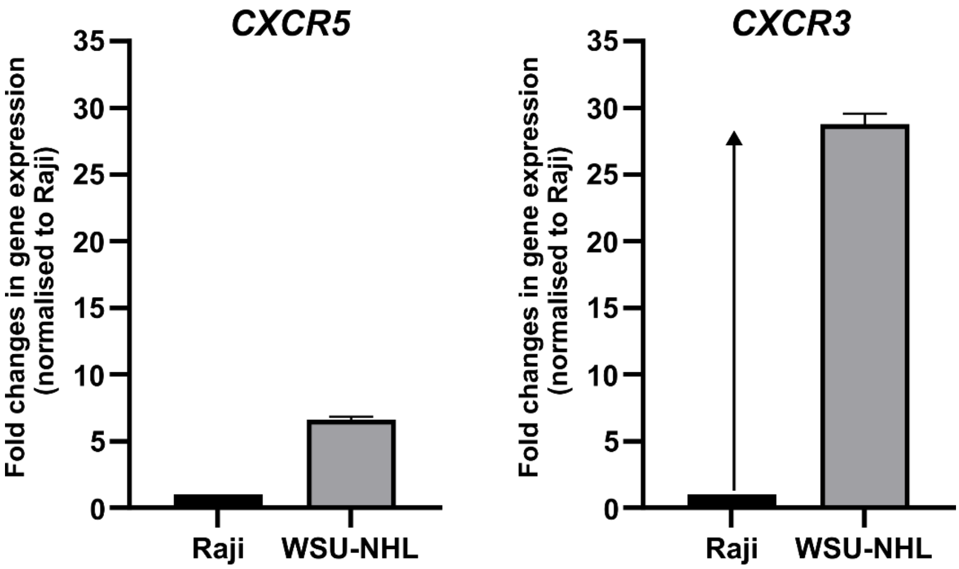

Figure S14. Relative mRNA expression of *CXCR3* and *CXCR5* genes in WSU-NHL and Raji cells determined by qPCR analysis. Data presented as means±SD from 2 independent experiments.

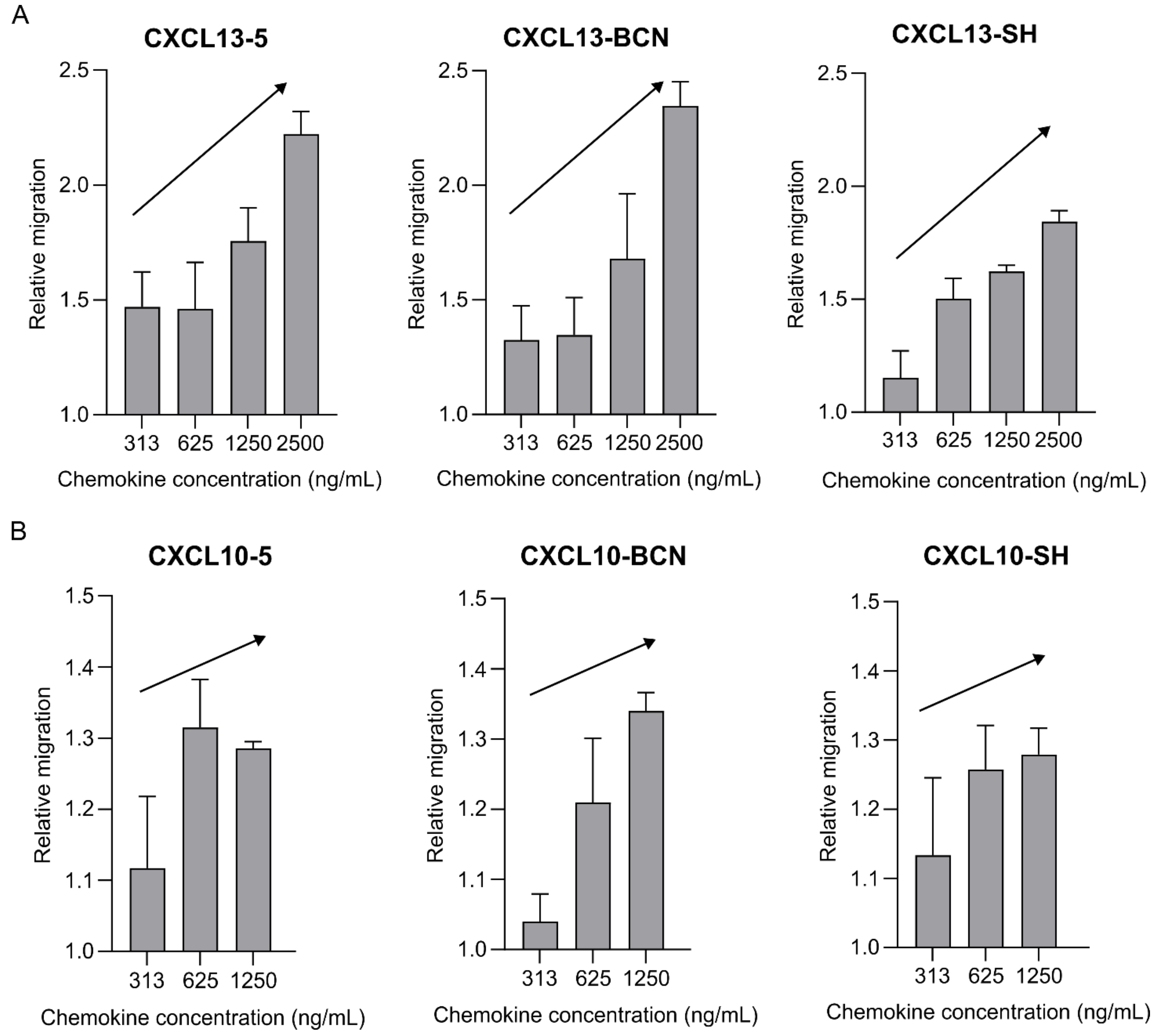

Figure S15. Transwell migration assays in WSU-NHL cells. Migrated cells were quantified (resazurin test) after incubation with hCXCL13 (A) and hCXCL10 (B) conjugates. Relative migration ratios were calculated by relating the fluorescence emission of stimulated cells and the fluorescence emission of cells with RPMI only (basal migration). Experiments were performed in triplicate and values are presented as means±SD.

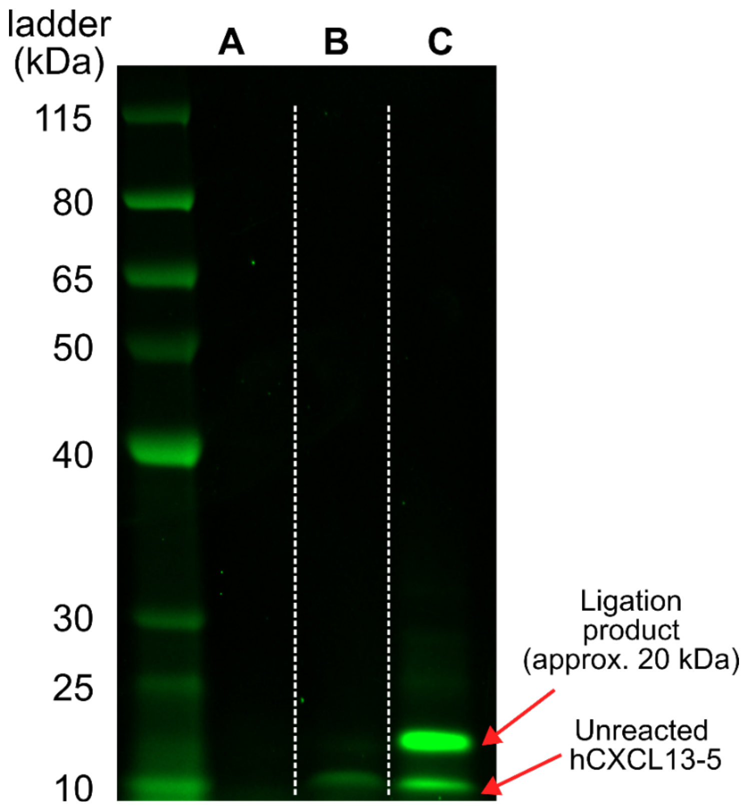

Figure S16. Representative SDS-PAGE gel of the reaction between **hCXCL13-5** and **hCXCL10-BCN**, displaying a fluorescent band corresponding to the chemokine ligation product by in-gel fluorescence analysis (λ_{exc}: 488 nm). Lanes A: **hCXCL10-BCN** (2 µg), B: **hCXCL13-5** (2 µg), C: **hCXCL10-BCN+hCXCL13-5** (2 µg + 2 µg).

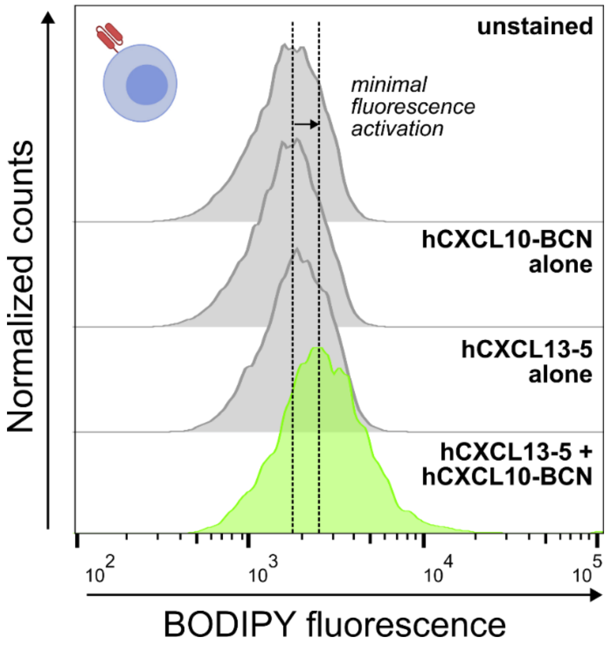

Figure S17. Dual chemokine ligation in Raji cells monitored by flow cytometry. Fluorescence histograms of Raji cells after incubation with **hCXCL13-5** (220 nM), **hCXCL10-BCN** (290 nM) or **hCXCL13-5+hCXCL10-BCN** using flow cytometry analysis.

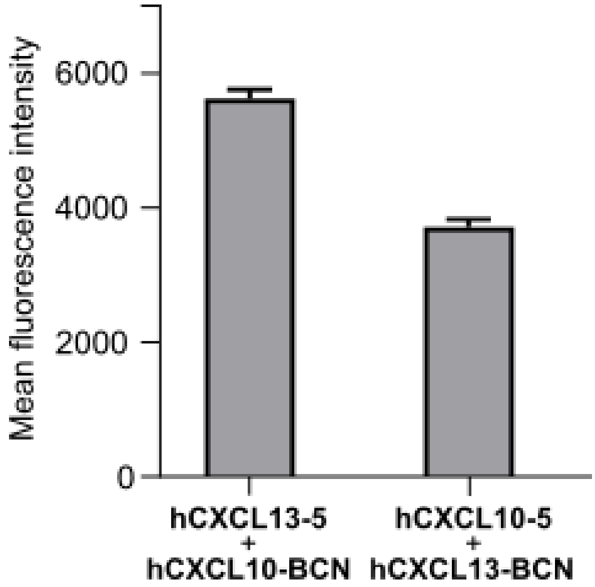

Figure S18. Flow cytometry analysis of drug-resistant WSU-NHL cells after staining for 30 min with two reagent combinations: left) **hCXCL13-5** (220 nM) + **hCXCL10-BCN** (290 nM), right) **hCXCL10-5** (220 nM) + **hCXCL13-BCN** (290 nM). Data presented as mean values±SD (n=3).

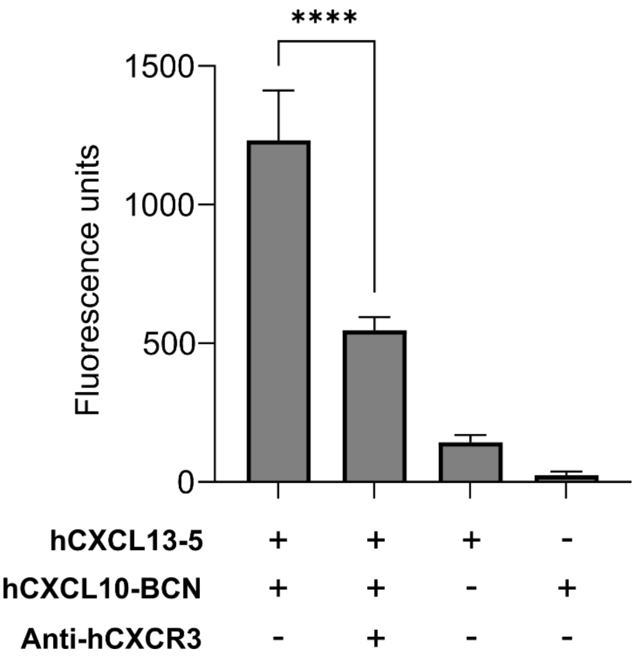

Figure S19. Flow cytometry analysis of dual chemokine ligation in WSU-NHL with **hCXCL13-5** and **hCXCL10-BCN** (both 500 ng/mL) in presence or absence of neutralizing anti-hCXCR3 antibody (20 µg/mL). Data presented as mean values±SD (n=3). P values determined by one-way ANOVA; **** for $p<0.0001$.

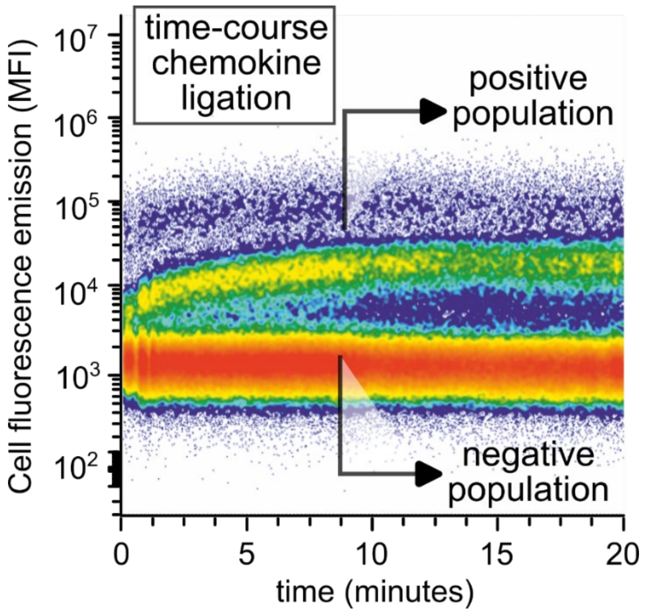

Figure S20. Time-course chemokine ligation analysis of the **hCXCL13-5** (235 nM) and **hCXCL10-BCN** (280 nM) in WSU-NHL B cells. Fluorescence emission was recorded for 20 min after addition of **hCXCL10-BCN**.

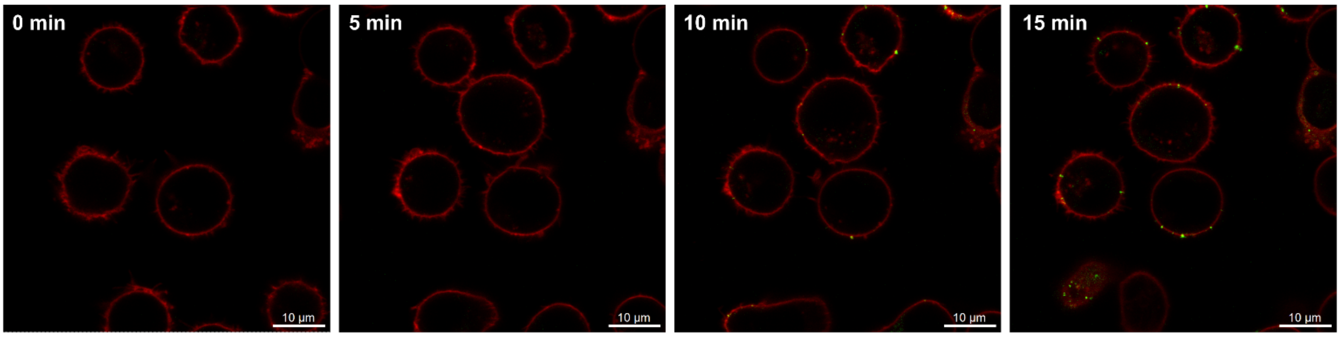

Figure S21. Representative time-course fluorescence microscopy images of Raji cells after incubation with **hCXCL10-BCN** (1 µM) before and after addition of **hCXCL13-5** (440 nM, green) under wash-free imaging conditions. Cells were co-stained with CellMask Deep Red (1:2000 dilution, red) as a membrane marker. Excitation wavelengths: 488 nm (for **hCXCL13-5**), 660 nm (for CellMask Deep Red). Scale bar: 10 µm.

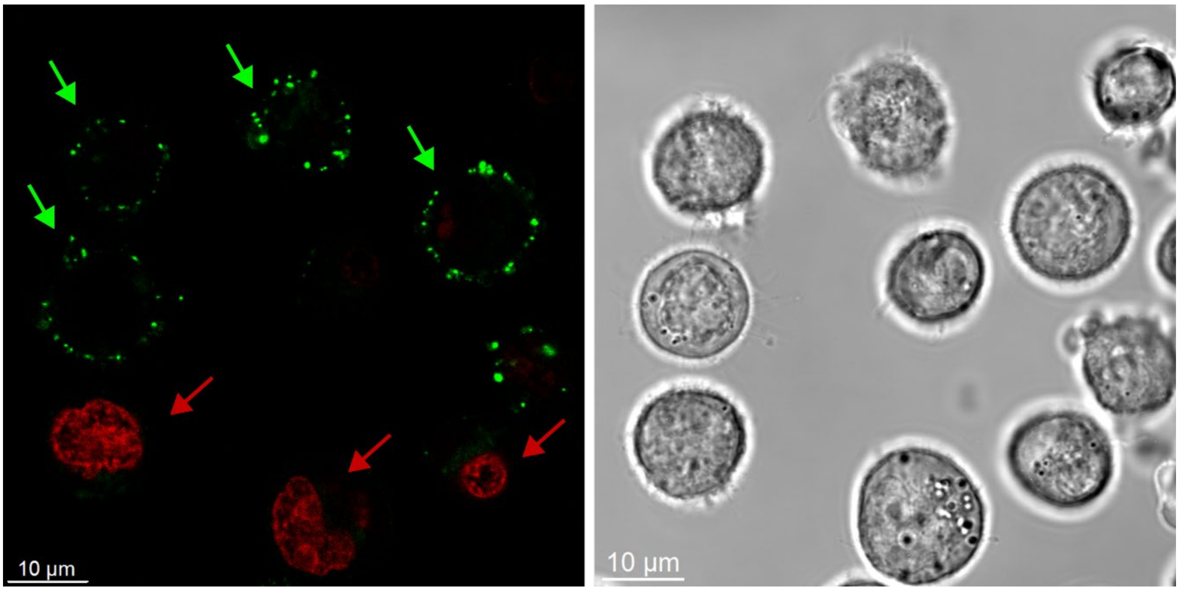

Figure S22. Representative brightfield and fluorescence microscopy images of co-cultured Raji (red arrows) and WSU-NHL cells (green arrows) incubated with **hCXCL10-BCN** (1 µM) and **hCXCL13-5** (440 nM, green). Raji cells were pre-stained with the nuclear dye DRAQ5 (5 µM) before mixing them with unstained WSU-NHL cells in a 1:1 ratio. Green fluorescence emission was observed exclusively in WSU-NHL cells. Excitation wavelengths: 488 nm (**hCXCL13-5**), 633 nm (DRAQ5). Scale bar: 10 µm.

3. Supplementary movies.

Movie S1. Time-lapse fluorogenic intracellular activation of compound **4** (10 μM, green) in MCF-7 cells after addition of excess BCN (100 μM). Cells were counterstained with DRAQ5 (5 μM, red). Movie recorded for 2 min and compressed at 8 fps.

Movie S2. Time-lapse fluorescence microscopy of Raji cells after incubation with **hCXCL13-5** (500 nM, green) followed by addition of BCN (50 μM). Cells were counterstained with DRAQ5 (5 μM, red). Movie recorded for 3 min and compressed at 8 fps.

Movie S3. Time-lapse fluorescence microscopy of WSU-NHL cells after incubation with **hCXCL13-5** (440 nM, green) followed by addition of **hCXCL10-BCN** (1 μM). Cells were incubated with Lysotracker Red (cyan, 1:1000 dilution) and CellMask Deep Red (red, 1:2000 dilution). Movie recorded for 15 min and compressed at 8 fps.

Movie S4. Time-lapse fluorescence microscopy of Raji cells after incubation with **hCXCL13-5** (440 nM, green) followed by addition of **hCXCL10-BCN** (1 μM). Cells were pre-incubated with Lysotracker Red (cyan, 1:1000 dilution) and CellMask Deep Red (red, 1:2000 dilution). Movie recorded for 15 min and compressed at 8 fps.

4. NMR Spectra

Compound **1** ($CDCl_3$)

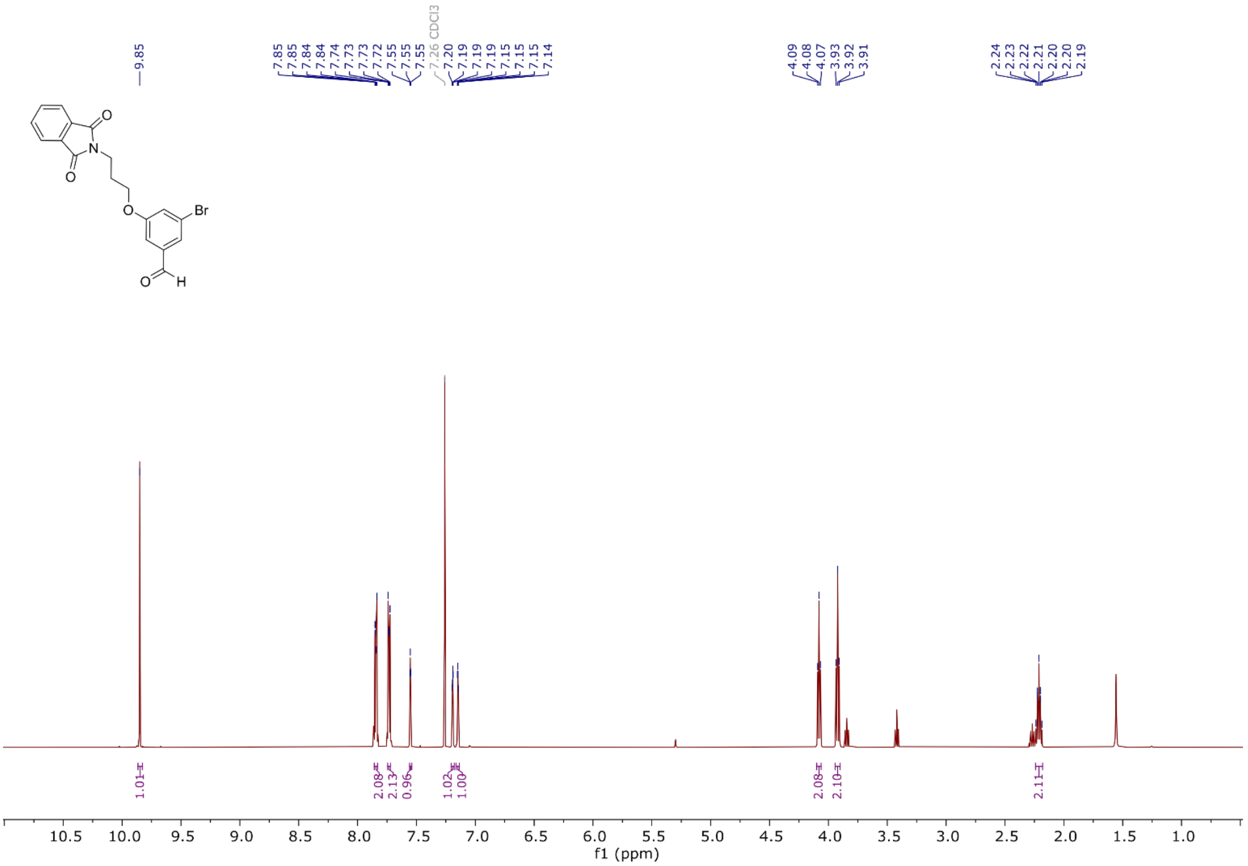

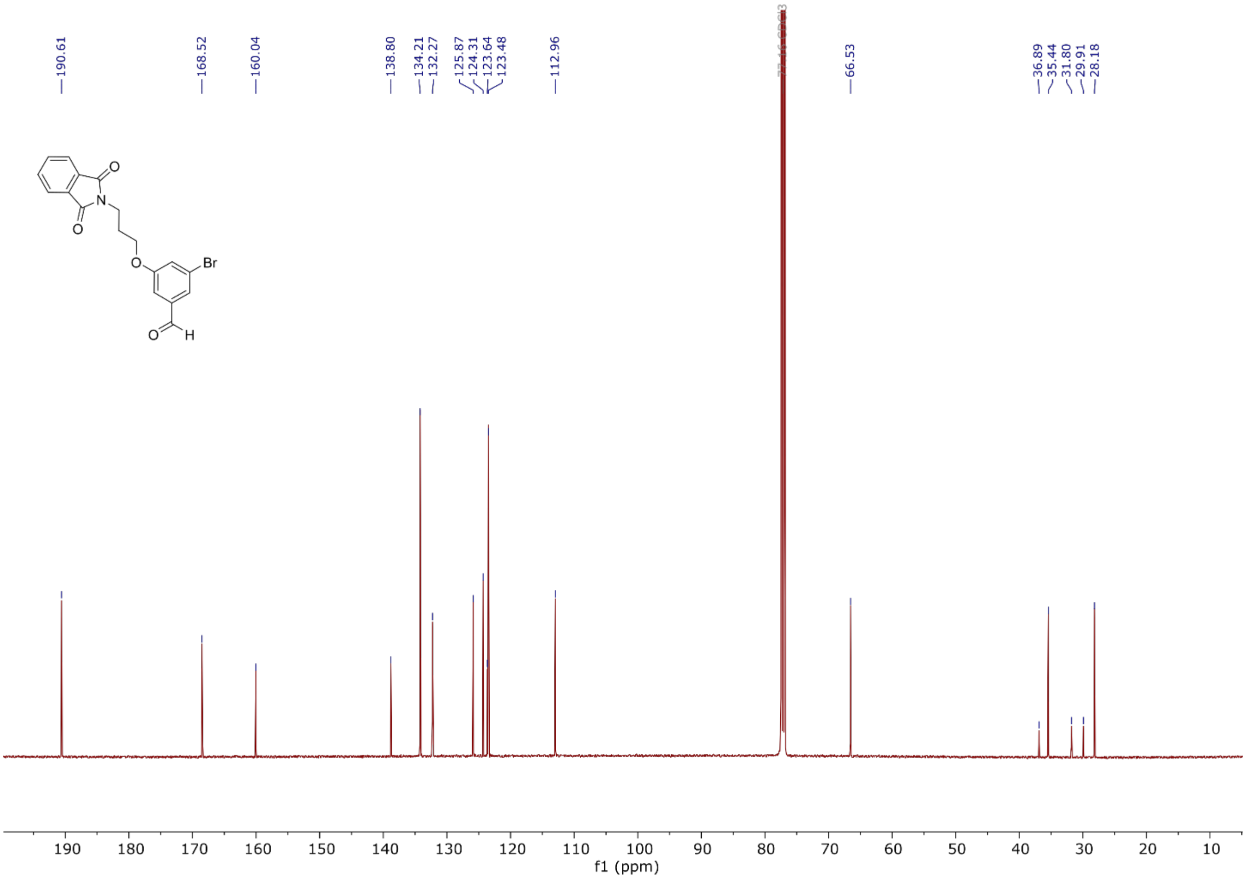

Compound **2** ($CDCl_3$)

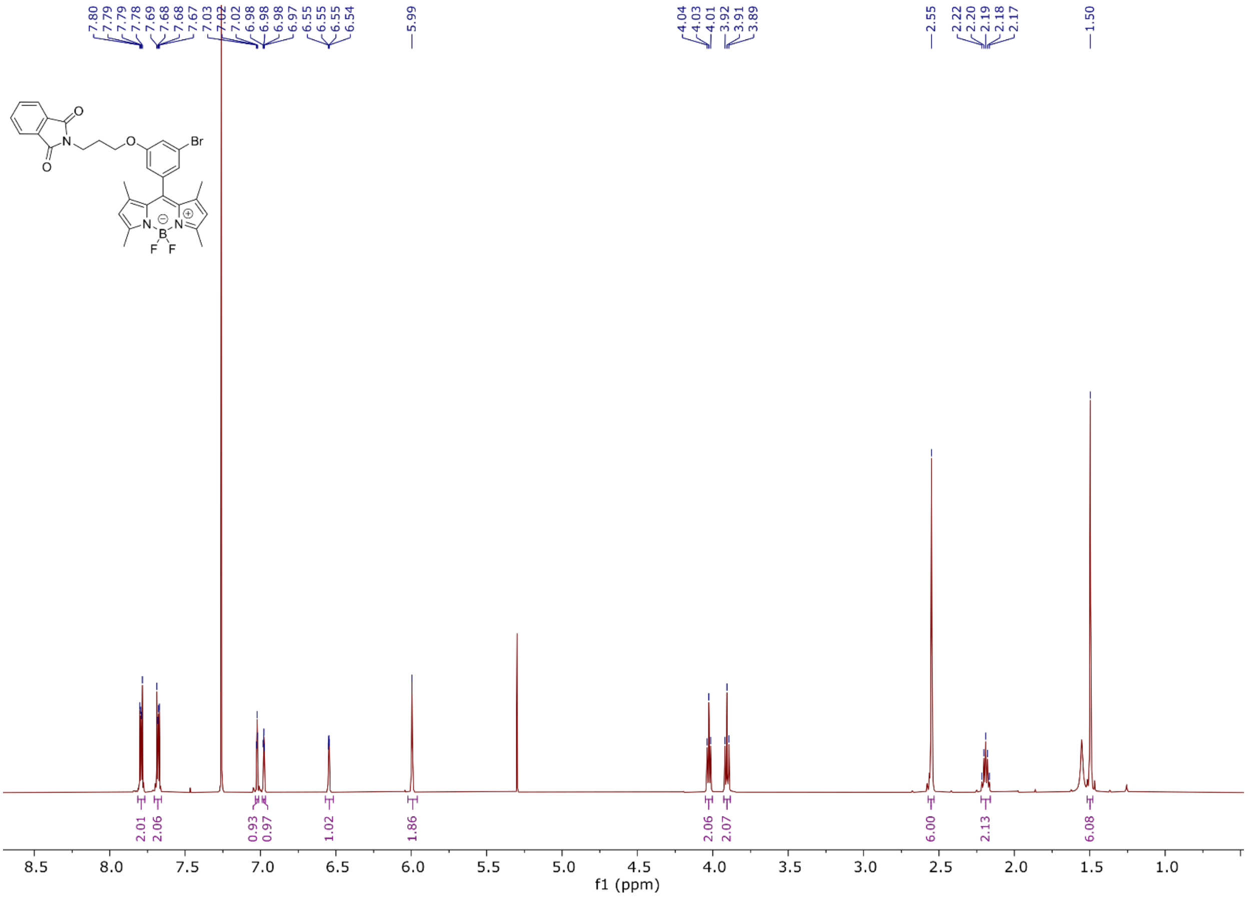

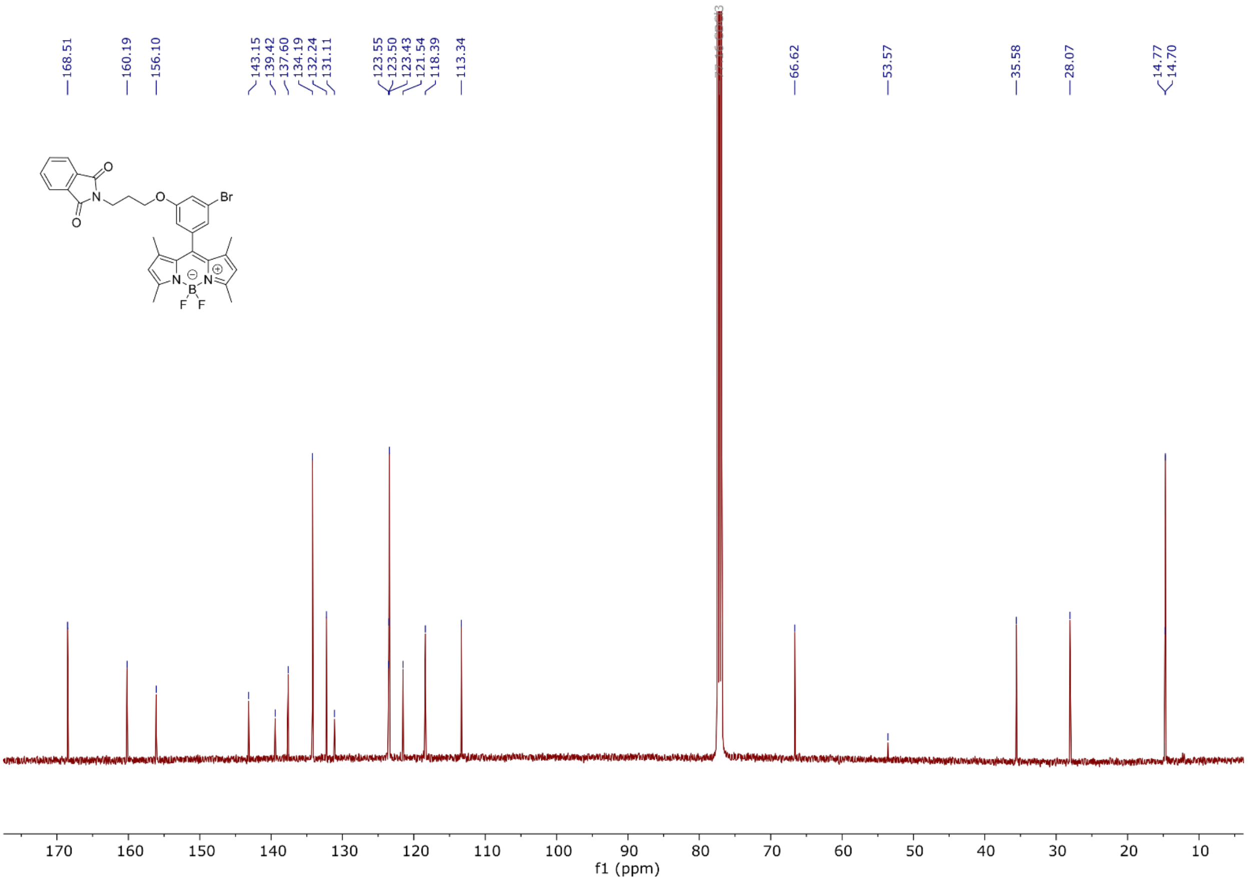

Compound **3** ($CDCl_3$)

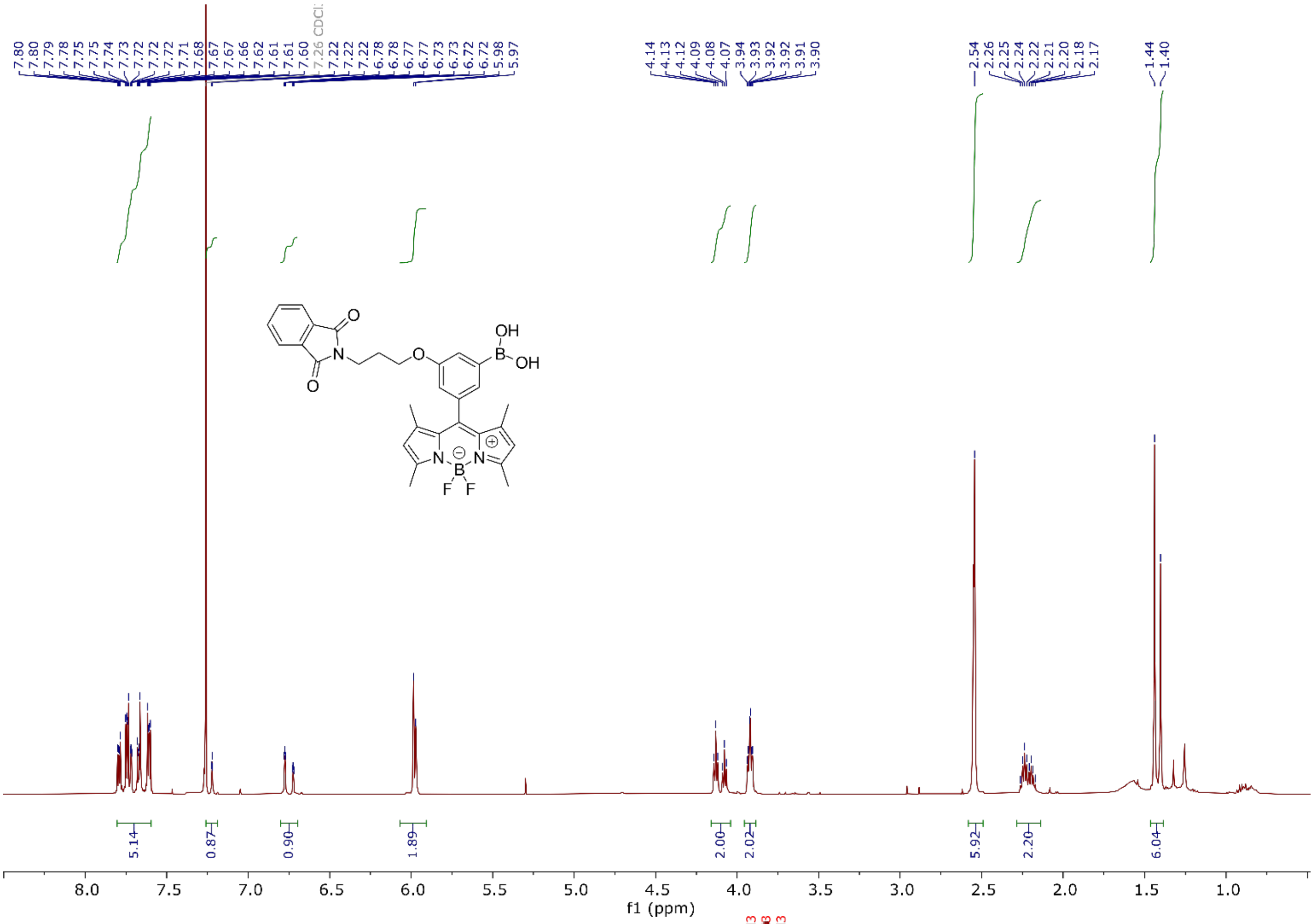

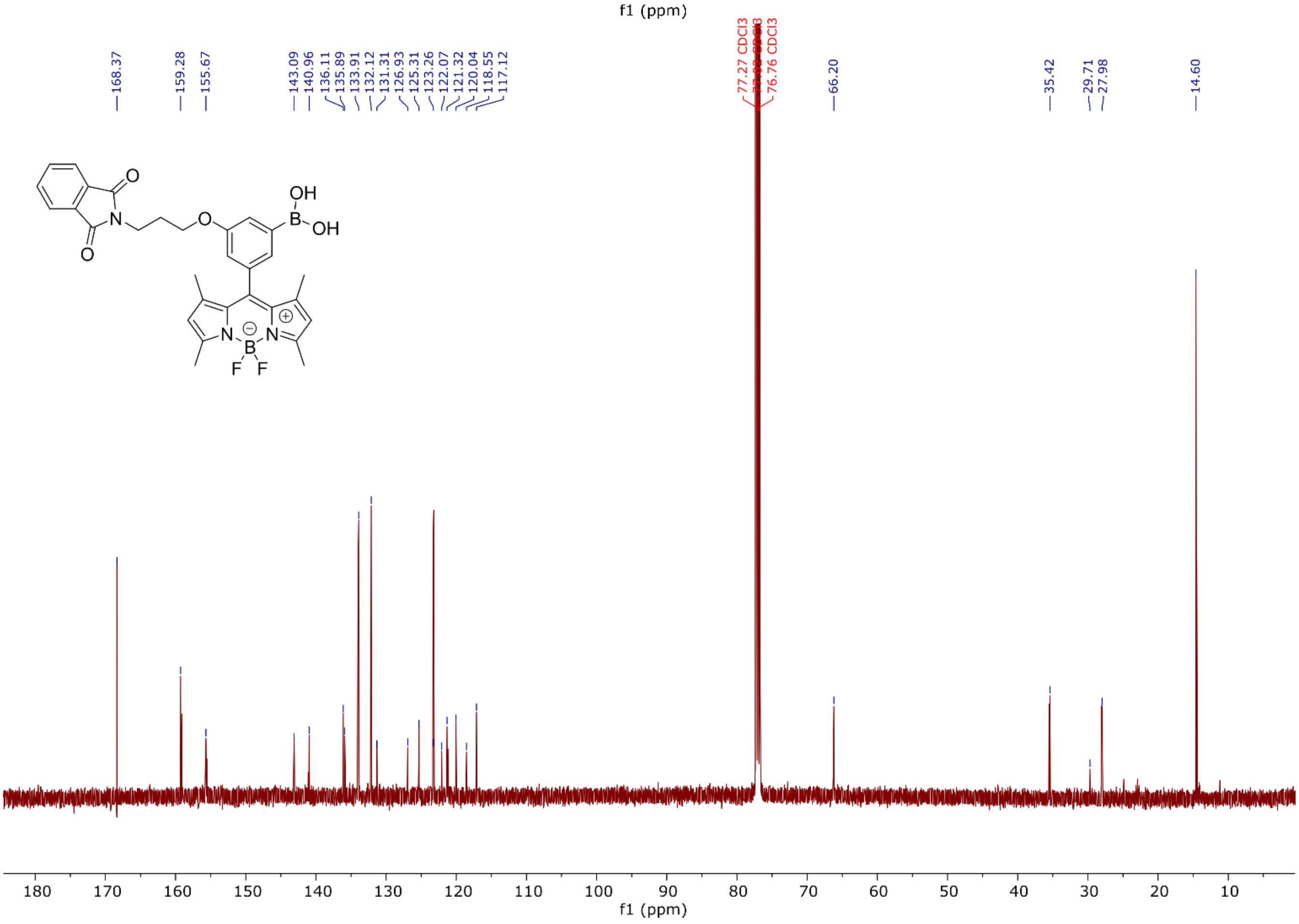

Compound **4** ($CDCl_3$)

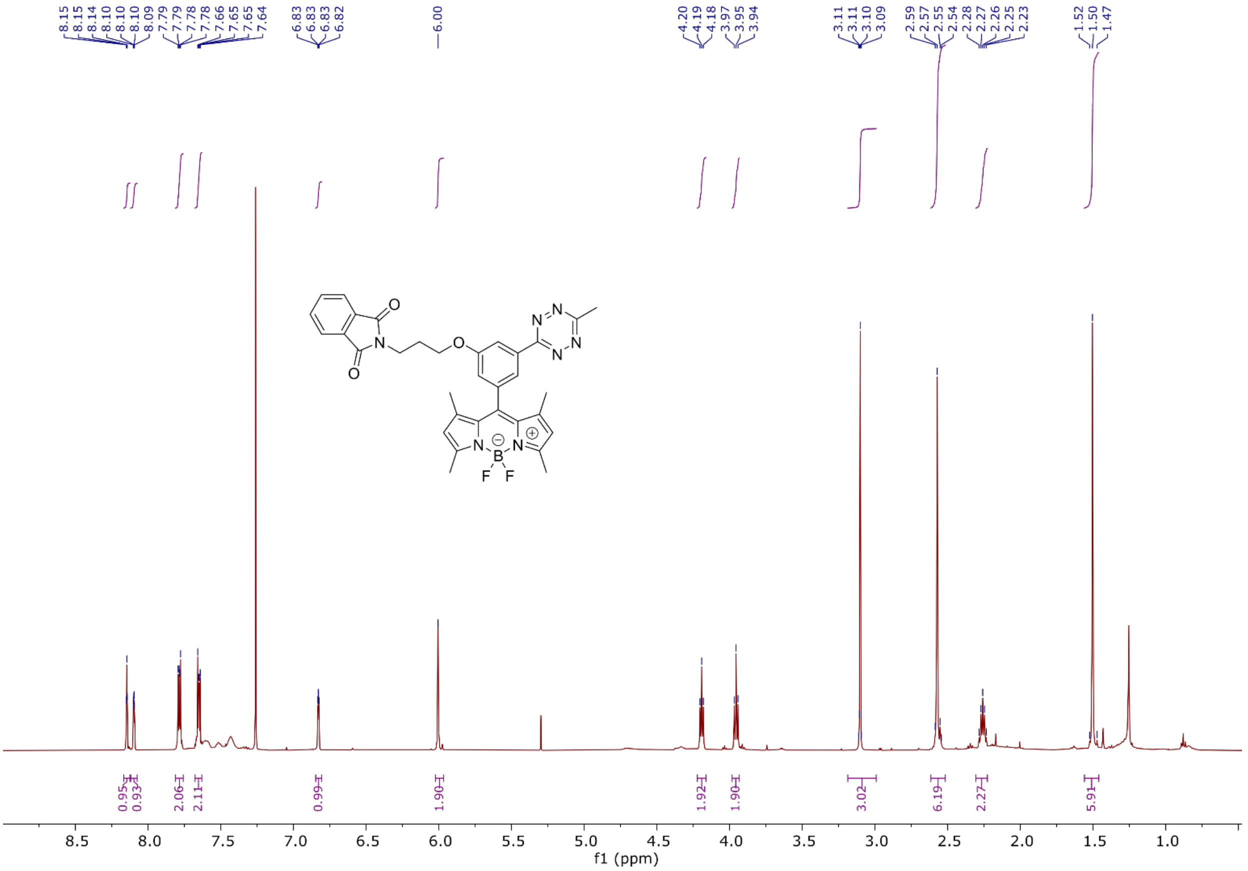

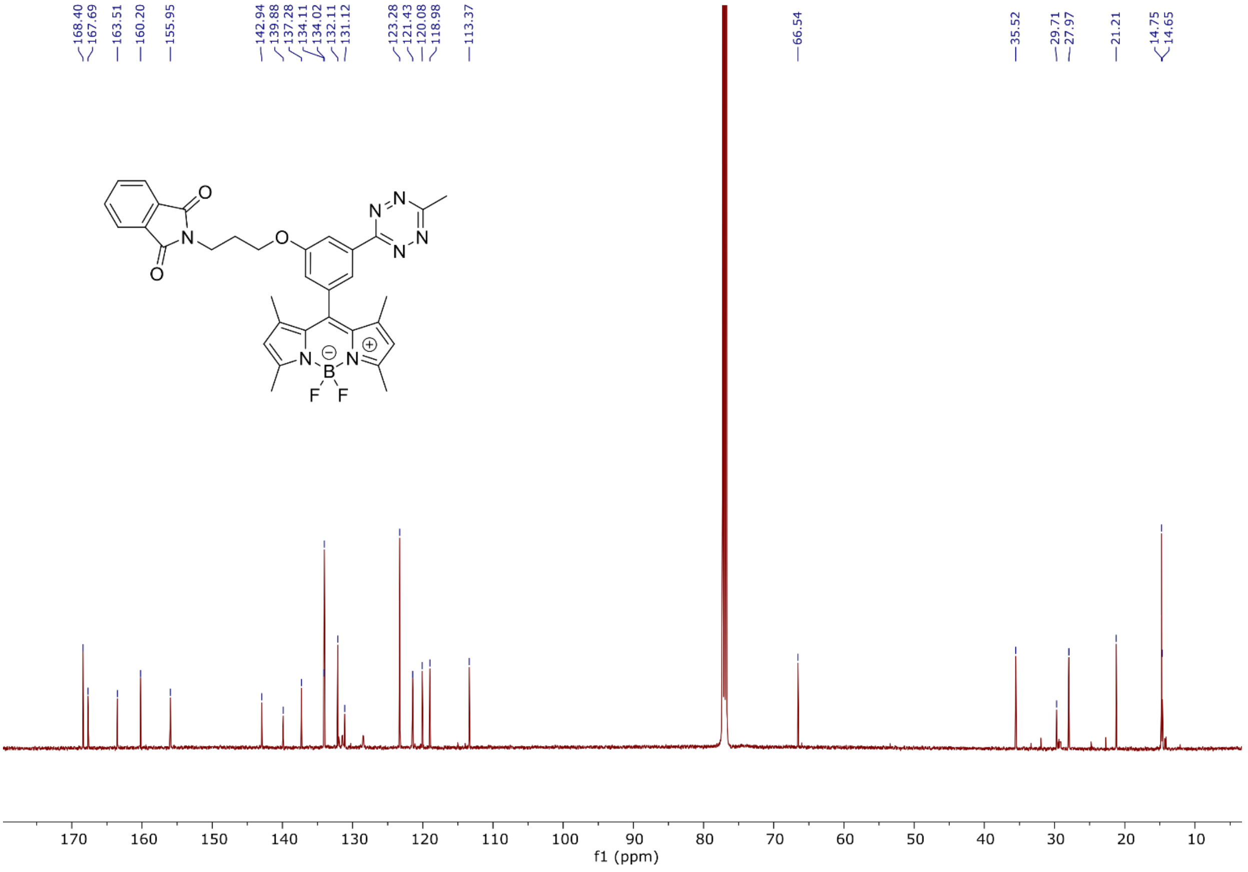

Supplement: Supplementary file 1 — ja4c12035_si_001.pdf [file ja4c12035_si_001.pdf]
